# Strategies for the Synthesis of 19-*nor*-Vitamin D Analogs

**DOI:** 10.3390/ph13080159

**Published:** 2020-07-22

**Authors:** Susana Fernández, Miguel Ferrero

**Affiliations:** Departamento de Química Orgánica e Inorgánica, Universidad de Oviedo, 33006 Oviedo, Asturias, Spain

**Keywords:** vitamin D, 19-*nor*-vitamin D_3_, 19-*nor*-vitamin D_2_, synthesis, modified analogs

## Abstract

1α,25-Dihydroxyvitamin D_3_ [1α,25-(OH)_2_-D_3_], the hormonally active form of vitamin D_3_, classically regulates bone formation, calcium, and phosphate homeostasis. In addition, this hormone also exerts non-classical effects in a wide variety of target tissues and cell types, such as inhibition of the proliferation and stimulation of the differentiation of normal and malignant cells. However, to produce these actions, supraphysiological doses are required resulting in calcemic effects that limit the use of this natural hormone. During the past 30 years, many structurally modified analogs of the 1α,25-(OH)_2_-D_3_ have been synthesized in order to find derivatives that can dissociate the beneficial antiproliferative effects from undesired calcemic effects. Among these candidates, 1α,25-(OH)_2_-19-*nor*-D_3_ analogs have shown promise as good derivatives since they show equal or better activity relative to the parent hormone but with reduced calcemic effects. In this review, we describe the synthetic strategies to obtain the 19-*nor*-D_3_ derivatives and briefly describe their physiological activities.

## 1. Introduction

**Figure 1:**
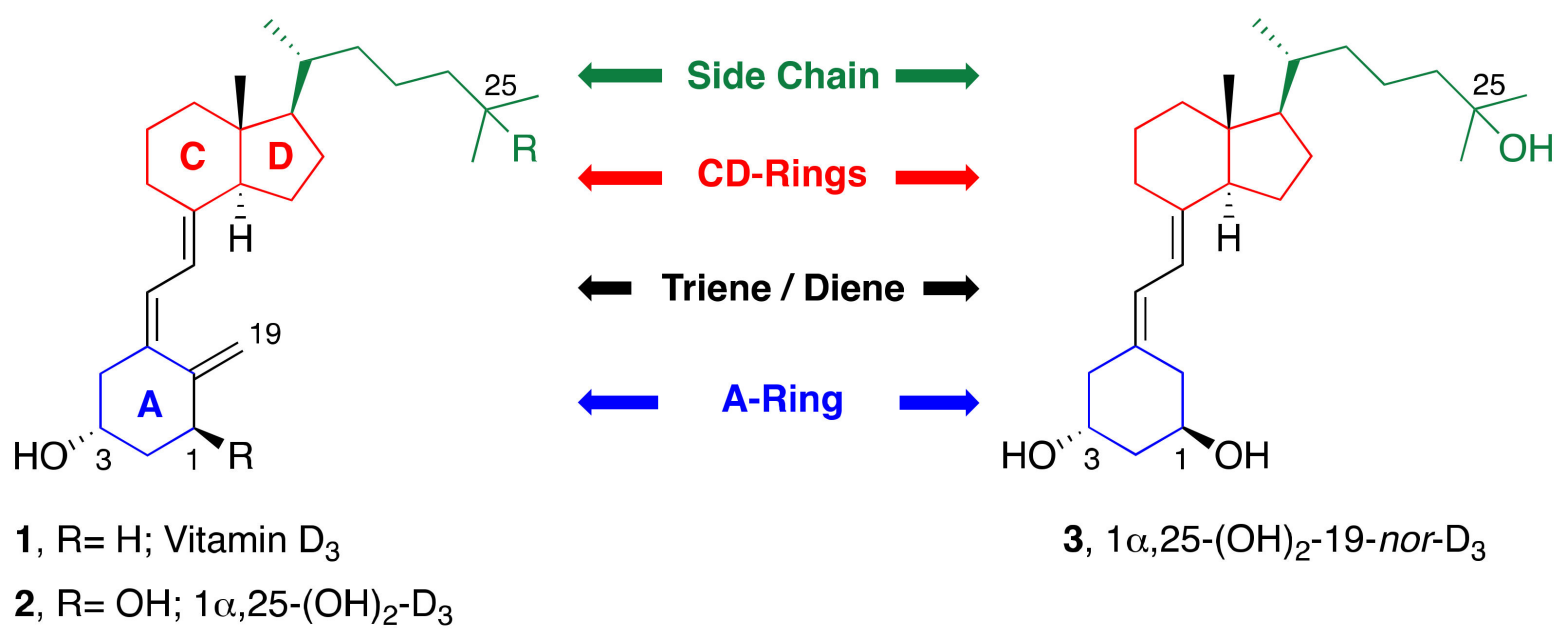
Structures of vitamin D_3_, its hormonally active form 1α,25-(OH)_2_-D_3_, and the 1α,25-(OH)_2_-19-*nor*-D_3_ analog.

The steroid hormone 1α,25-dihydroxyvitamin D_3_ [1α,25-(OH)_2_-D_3_] (**2**, Figure 1) is the active form of vitamin D_3_ (**1**), which can be synthesized in the skin or obtained from dietary sources [1]. Its primary physiological role is in the regulation of bone formation, calcium, and phosphate homeostasis [2]. In addition, it is a potent differentiator and growth inhibitor of several types of cancer cells. These observations have suggested its potential therapeutic application, but supraphysiological doses are required, resulting in calcemic effects that limit the use of this natural hormone [3,4].

In order to overcome this limitation, many structurally modified analogs of 1α,25-(OH)_2_-D_3_ have been synthesized in the past three decades [5,6,7,8,9,10,11]. Among these modifications, the removal of carbon C19 on the A-ring results in selective activities, inducing potent differentiation and growth inhibition of tumor cells, without a parallel increase in bone calciotropic activity. As a consequence of this structural characteristic, these analogs are more stable since they cannot undergo a [1,7] hydrogen sigmatropic shift to transform into their respective previtamin forms. The objective of this review is to collect the synthetic strategies that have been employed to obtain 1α,25-(OH)_2_-19-*nor*-D_3_ (**3**) derivatives.

The structurally dynamic hormone **2** presents four parts: An A-ring, conjugated triene (*seco* B-ring), CD-ring, and side chain. The main characteristic of 19-*nor* analogs is the absence of the C19 methylene group, so the “triene” part of these analogs is altered to just a diene moiety.

**Figure 2:**
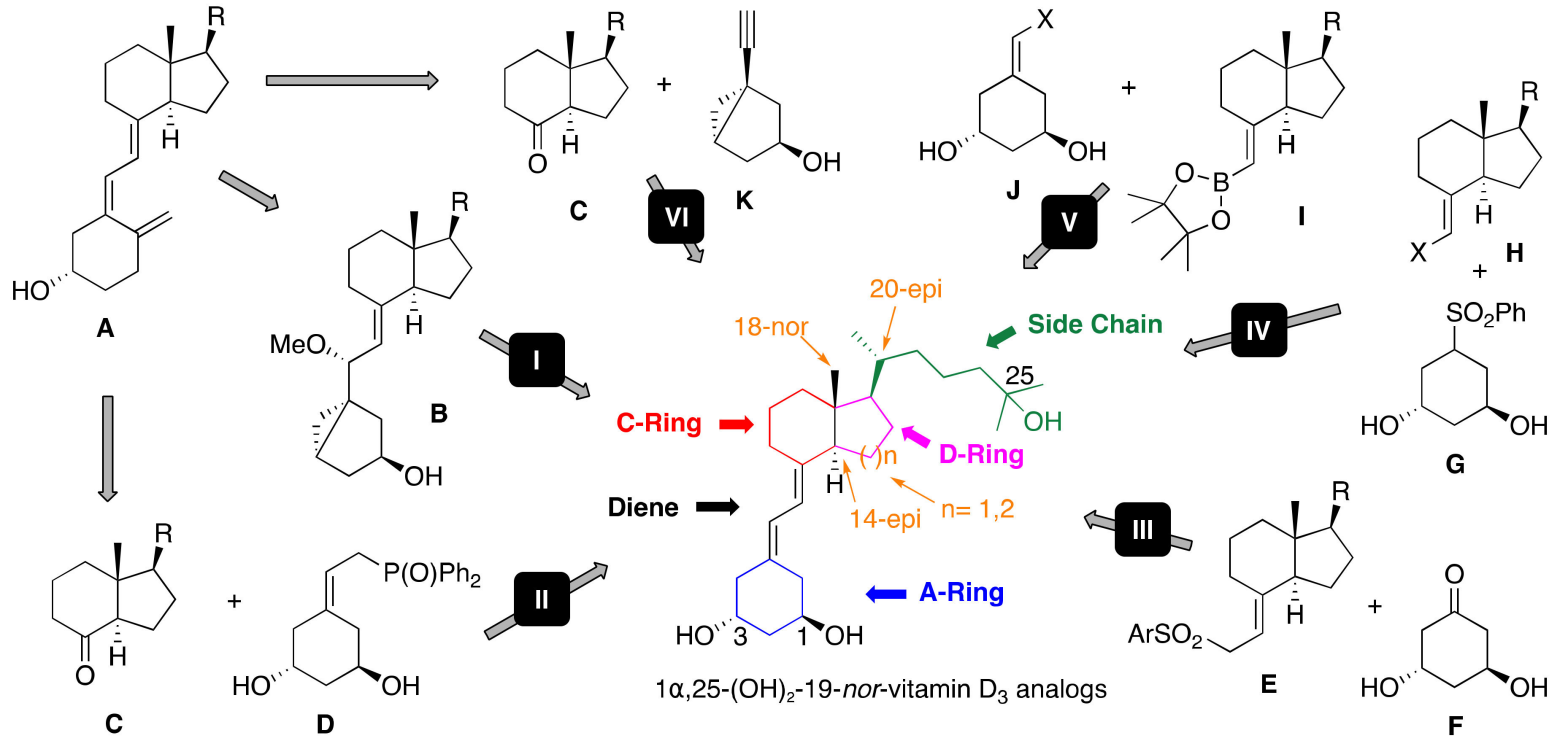
Major synthetic routes to prepare 1α,25-(OH)_2_-19-*nor*-vitamin D_3_ analogs.

A summary of the major synthetic routes utilized to prepare 1α,25-(OH)_2_-19-*nor*-vitamin D_3_ analogs is depicted in Figure 2. Method I is related to the direct modifications in the *seco*-B steroid precursors of type **A**. It is a linear approach to synthesizing new 19-*nor*-vitamin D analogs via a cyclovitamin intermediate **B**. The main characteristic of the synthesis, its linearity, is a great disadvantage since only in a few modifications can be used, and the global yields are lower than that obtained when convergent strategies are utilized. Method II constructs the diene moiety of 19-*nor*-vitamin D using the approach developed by Lythgoe [12] through a Wittig–Horner coupling reaction of a phosphine oxide A-ring synthon precursor **D** with a CD-ring/side chain fragment **C**, generally obtained from the oxidation of easily available vitamin D_2_ or vitamin D_3_ (**A**). It is the most used method to produce any kind of vitamin D analogs due to the versatility provided by using convergent synthesis. The limitation of this route, the tedious synthesis of the A-ring fragment **D**, has been circumvented by the development of several pathways to synthesize the appropriate A-ring precursors from natural starting materials. Another convergent approach to synthesizing 19-*nor* derivatives is through Julia olefination (Method III), which consists of the addition of a CD-ring/side chain sulfonyl-stabilized carbanion of **E** to an A-ring ketone **F**, followed by elimination to form an alkene. It has also been applied to the reverse synthon precursors, that is, the sulfone of the A-ring **G** and a vinyl halide of the CD-ring/side chain **H** (Method IV). To construct the diene part of the molecule, approaches based on the Suzuki–Miyaura reaction between the CD-ring boronate ester **I** and vinyl halides of the corresponding A-ring fragment **J** have been used (Method V). Method VI, based on the initial studies of Mazur, involves a bicyclo[3.1.0]hexane intermediate **K**, which is coupled with the CD ring/side chain fragment **C**. Solvolysis of an intermediate similar to **B** gives rise to vitamin D metabolites and analogs.

The information presented in this article is structured as follows. First, the syntheses of 19-*nor* vitamin D (D_2_/D_3_) with no other modifications in their structure compared to vitamin D, except for the absence of the methyl group at C19, are described. Then, different methods are shown for the syntheses of analogs with modifications in the A ring, followed by those in the diene part, the CD-ring, and, finally, the side chain. For cases of analogs with more than one modification, the methods are classified as one of the four categories indicated above and then subclassified by the additional modification(s) following the same order.

## 2. Synthesis of 1α,25-(OH)_2_-19-*nor*-Vitamin D

**Scheme 1:**
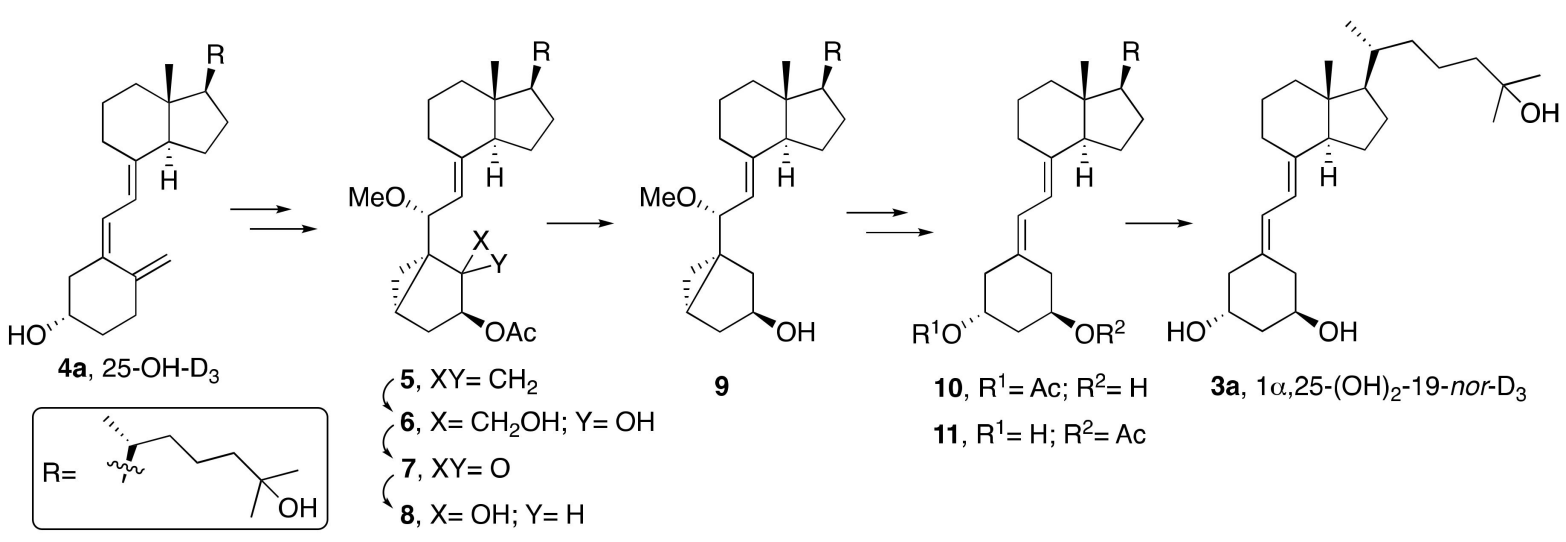
The first synthesis of 1α,25-(OH)_2_-19-*nor*-vitamin D_3_.

There are several synthetic approaches to synthesizing 1α,25-(OH)_2_-19-*nor*-D_3_. Thus, DeLuca and co-workers [13] described the synthesis of **3a** via oxidative degradation of the 1α-hydroxycyclovitamin intermediate **9** (Scheme 1). The first step consists of the transformation of 25-hydroxyvitamin D_3_ (**4a**) into 1α-acetoxy-25-OH-3,5-cyclovitamin D_3_ (**5**). Compound **5** was oxidized to form a mixture of diols **6**, which was subjected to diol cleavage to obtain the 10-oxo derivative **7**. The latter was reduced to produce an epimeric mixture of alcohols **8**. The mixture was then mesylated and reduced to generate 1α,25-(OH)_2_-19-*nor*-cyclo-D_3_ **9**, which underwent cycloreversion with acetic acid to **10** and **11**. Final hydrolysis of the acetates **10** and **11** resulted in the final product 1α,25-(OH)_2_-19-*nor*-D_3_ (**3a**). This procedure allowed the authors to prepare **3a** for the first time, which showed a combination of high potency induction of differentiation and very low bone calcification activity.

**Scheme 2:**
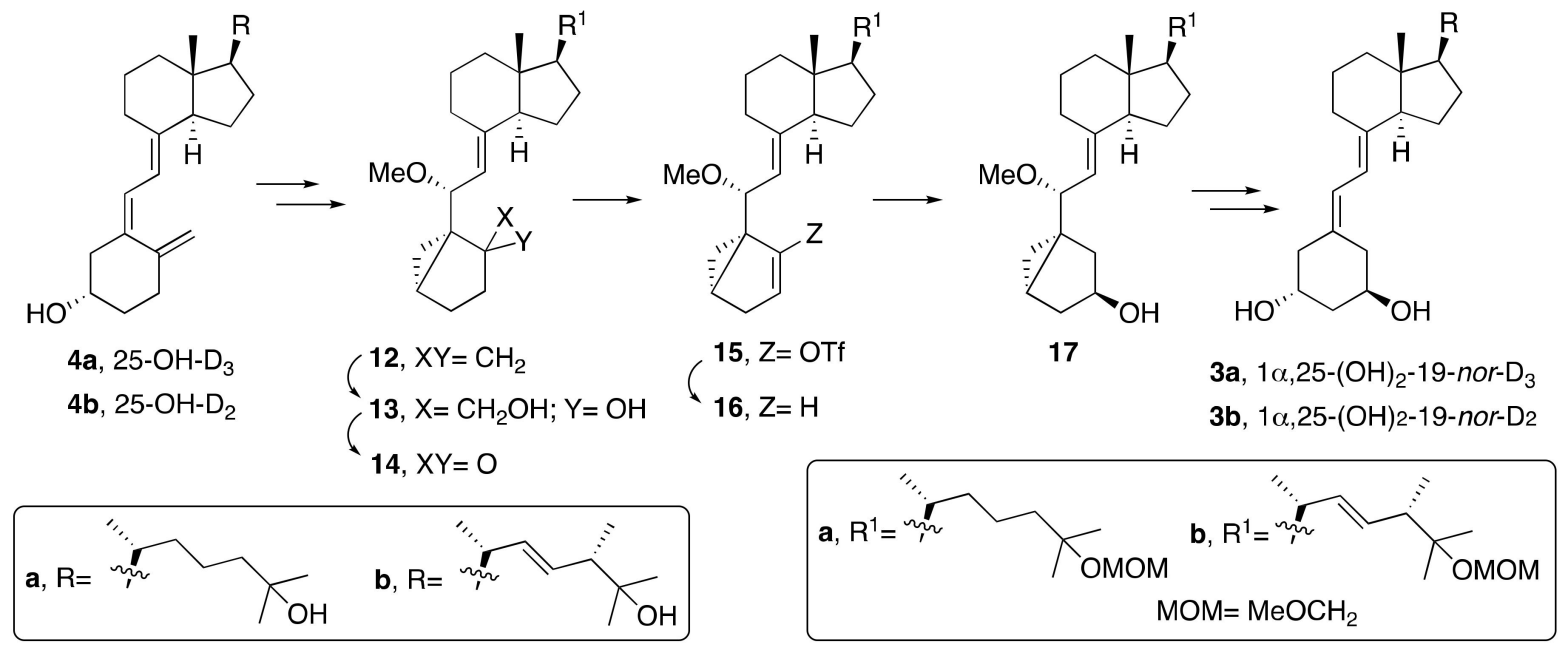
Routes to 1α,25-(OH)_2_-19-*nor*-vitamin D using regio- and stereoselective hydroboration.

On the other hand, Nagasawa and coworkers [14] reported the preparation of 1α,25-(OH)_2_-19-*nor*-D (**3a**) and (**3b**) from 25-OH-D (**4a** and **4b**) (Scheme 2). Compounds **4a** and **4b** were obtained through a bioconversion method. The key intermediate is 10-keto-3,5-cyclovitamin D **14** formed in a similar manner to that previously described. Conversion of **14a**-**b** to **3a**-**b** was carried out using a regio- and stereoselective hydroboration reaction with the olefin **16** with the bulky reagent 9-BBN, affording the 1α-hydroxy-3,5-cyclovitamin D derivatives **17** exclusively. Cycloreversion of **17** with acetic acid followed by hydrolysis of the resulting acetate produced a diol, which was deprotected at C25 with CSA to produce 1α,25-(OH)_2_-19-*nor*-D_3_ (**3a**) and -D_2_ (**3b**), respectively. Paricalcitol (**3b**) was approved in the late 1990s for the treatment and prevention of secondary hyperparathyroidism associated with chronic renal failure.

**Scheme 3:**
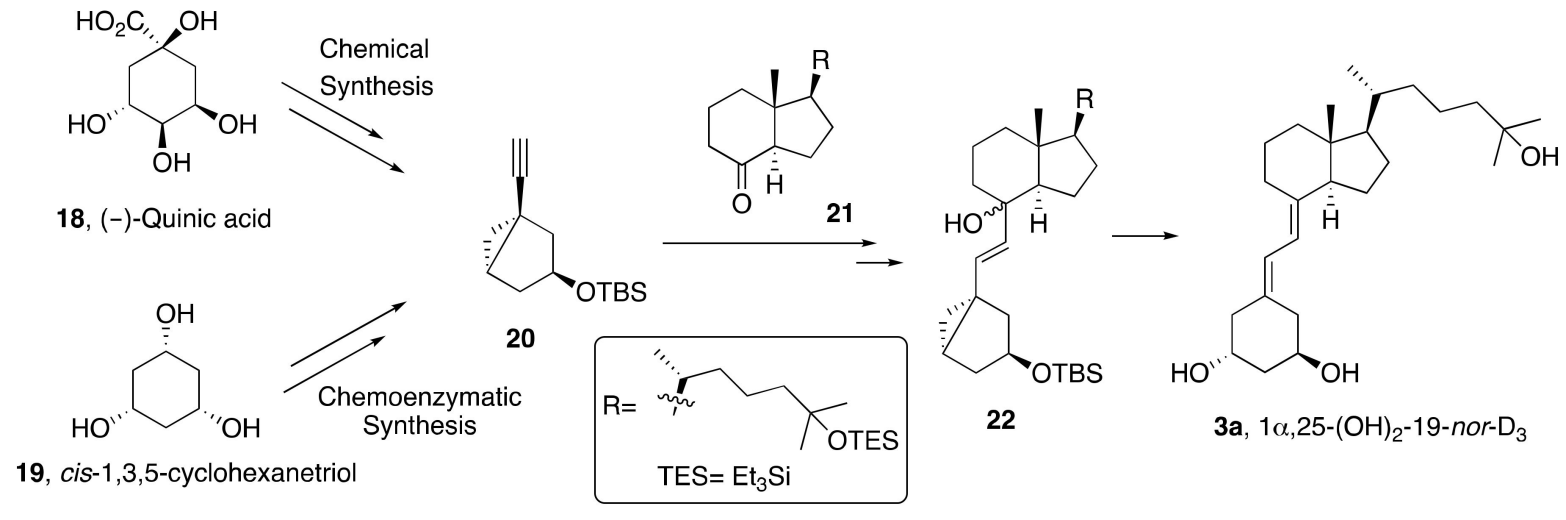
Convergent routes to 1α,25-(OH)_2_-19-*nor*-vitamin D_3_.

An alternative synthesis involving a new 19-*nor* A-ring precursor **20** (Scheme 3) and based on the well-known sigmatropic rearrangement of cyclopropylic alcohols into homoallylic alcohols was reported by Vandewalle and co-workers [15]. They proposed two alternative routes to the A-ring precursor **20**. The first chemical approach began with (−)-quinic acid (**18**) and centered on the removal of the C1 and C4 hydroxyl groups and on intramolecular alkylation of an ester-enolate to generate the bicyclic product precursor of **20**. Then, the ester was transformed into an aldehyde that was finally converted into an alkyne using dimethyl diazomethylphosphonate. They also developed a chemoenzymatic route to the A-ring **20** starting from *cis*-1,3,5-cyclohexanetriol (**19**), easily available upon catalytic hydrogenation of phloroglucinol. In order to obtain the vitamin D analog, the lithiated alkyne **20** was reacted with a ketone **21** to generate the propargylic alcohol, which was subsequently reduced to the (*E*)-allylic alcohol **22**. Acid-catalyzed solvolysis of **22** produced 1α,25-(OH)_2_-19-*nor*-D_3_ (**3a**). The *E*-geometry of the 7,8-double bond arises from the steric hindrance exerted by the D-ring. Thus, because of the C_2_-symmetry nature of the A-ring, only the desired geometry of **3a** was observed.

**Scheme 4:**
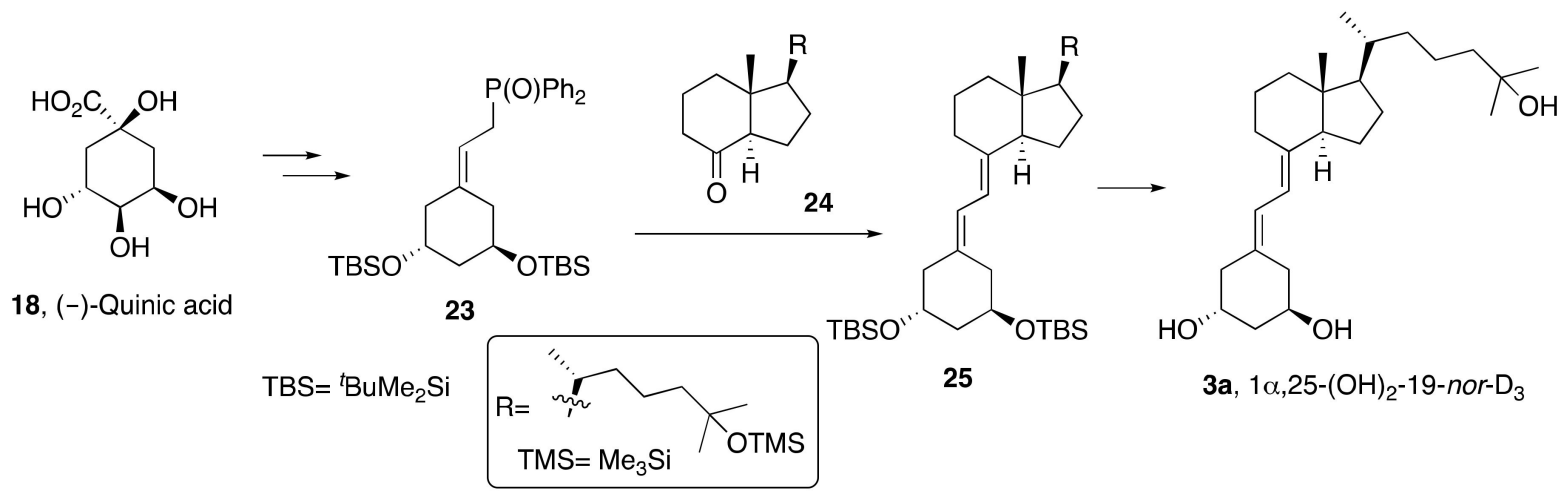
Large-scale preparation of 1α,25-(OH)_2_-19-*nor*-vitamin D_3_.

DeLuca’s research group [16] described an alternative approach to preparing 1α,25-(OH)_2_-19-*nor*-D_3_ (**3a**) that consists of a convergent route more suitable for large-scale preparations (Scheme 4). The new synthesis entails the independent preparation of a phosphine oxide A-ring precursor **23** and the CD-ring/side chain ketone **24** and their eventual condensation through a Wittig–Horner reaction to obtain the 19-*nor*-vitamin D derivative **25**, which, after deprotection, yielded the desired compound **3a**. They chose (−)-quinic acid (**18**) as the starting material since it features the correct hydroxy stereochemistry and is commercially available.

**Scheme 5:**
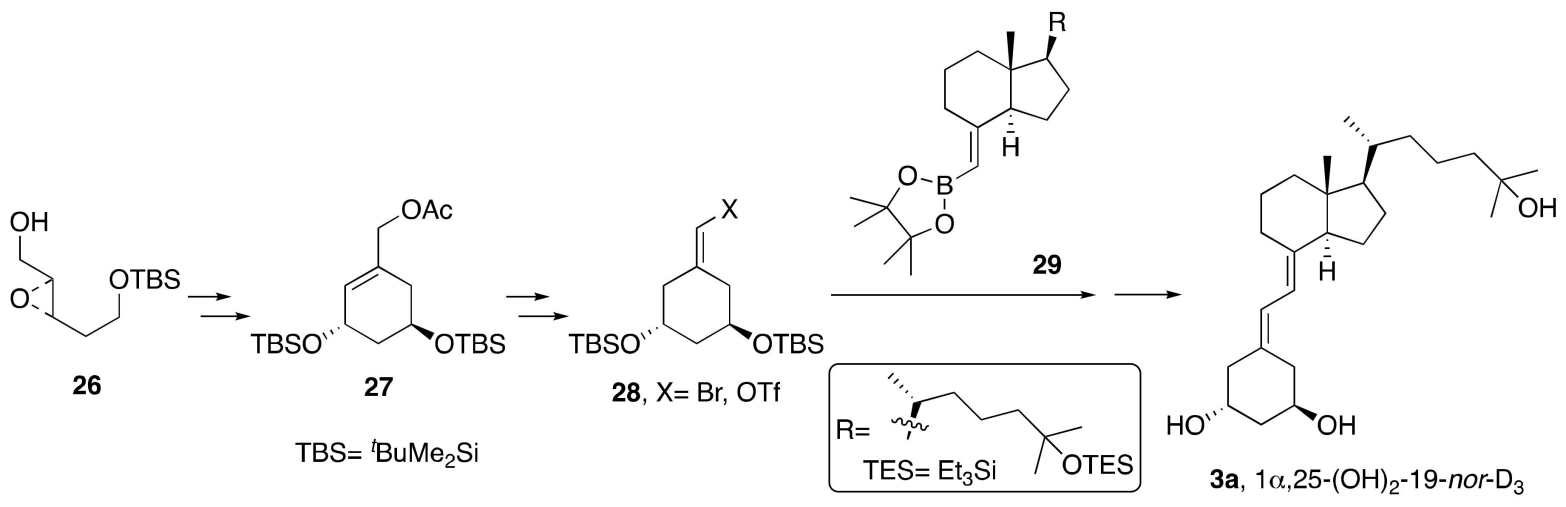
Use of ring-closing metathesis to synthesize 1α,25-(OH)_2_-19-*nor*-vitamin D_3_.

Nagasawa’s group described [17] a new synthetic method to obtain A-ring synthons **28** (Scheme 5) based on ring-closing metathesis from a linear precursor, and then they applied it to synthesize 1α,25-(OH)_2_-19-*nor*-D_3_ (**3a**). This strategy enabled the preparation of an A-ring with a more flexible substitution pattern. For the synthesis of A-ring precursors **28** they chose cyclohexene **27** as an intermediate, which was obtained from the corresponding chiral epoxide alcohol **26**, derived from 1,3-propanediol. These A-rings **28** were used to carry out the coupling with the appropriate functionalized CD-ring/side chain fragment **29** to produce 1α,25-(OH)_2_-19-*nor*-D_3_ (**3a**).

## 3. Synthesis of A-Ring-Modified 1α,25-(OH)_2_-19-*nor*-Vitamin D

**Scheme 6:**
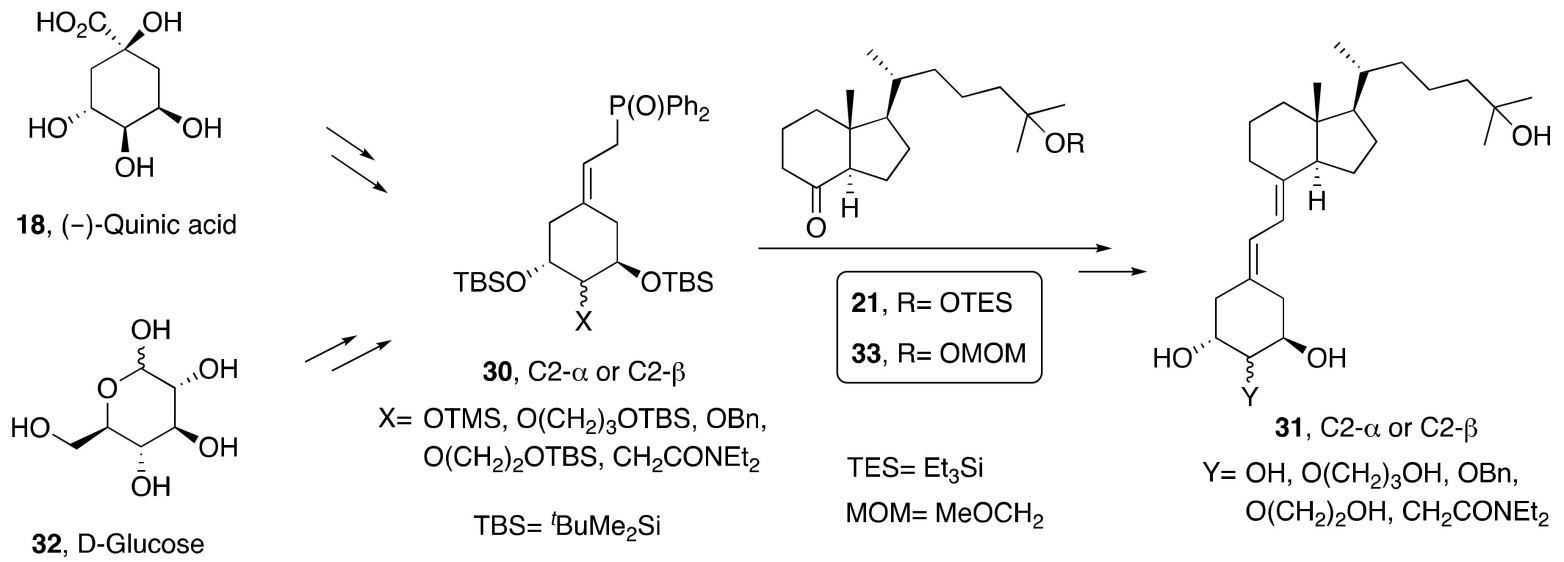
Preparation of C2-substituted 1α,25-(OH)_2_-19-*nor*-vitamin D_3_.

DeLuca and co-workers [18] reported the synthesis of 1α,2α/β,25-(OH)_3_-19-*nor*-D_3_ and their alkoxy derivatives (**31**, Scheme 6). The objective was to synthesize new analogs with potential applications as drugs for osteoporosis but with no calcemic effects. They were prepared through a convergent route, starting from (−)-quinic acid (**18**) and 25-hydroxy-protected Grundmann’s ketone (**21**) followed by the Wittig–Horner coupling approach using the phosphine oxide precursors **30**. Since an alkylation of the 2-hydroxy group was performed at the end of the synthesis, several analogs with different alkoxy substituents at the 2-position were conveniently obtained. On the basis of the same synthetic approach, Shimizu, Yamada, and co-workers [19] synthesized C2-alkoxy-substituted analogs **31** by a new sequence starting from d-glucose (**32**) as a chiral template to prepare A-ring synthons **30**. Transformation of the pyranoside ring into the A-ring carbocycle was achieved by the Pd-catalyzed Ferrier rearrangement. Using this new methodology, analogs **31** were obtained in a novel cost-effective method by coupling of the A-ring precursors **30** with the CD-ring/side chain moiety **33**.

**Scheme 7:**
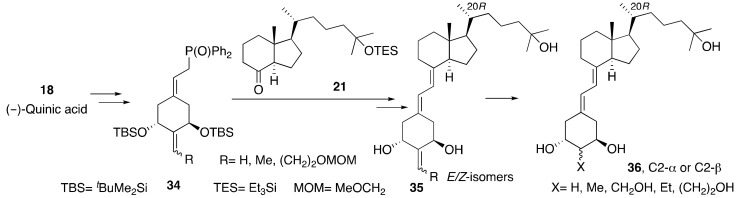
Preparation of C2-substituted 1α,25-(OH)_2_-19-*nor*-vitamin D_3_ from quinic acid.

The results of previous studies on 19-*nor* analogs of the hormone 1α,25-(OH)_2_-D_3_ (**2**) indicate that substituents on C2 completely change the biological potency of these derivatives since they alter the orientation of the 1α-hydroxyl group. Thus, DeLuca’s group reported new highly active isomers **36** (Scheme 7) via methylene [20] or ethylene (both *E* and *Z* geometrical isomers) [21] intermediates **35**, prepared in a convergent manner from (−)-quinic acid. The synthetic pathway involved a Wittig–Horner coupling of the corresponding A-ring phosphine oxides **34** with the corresponding 25-hydroxy protected Grundmann’s ketone **21**. Notably, the analogs **35** possessed C2-substituents as *E*- and *Z*-isomers of 3′-hydroxypropylidene, and a derivative of the former compound possessed a 3′-(methoxymethyl)propylidene [22]. In vivo tests revealed that the calcemic activity of all analogs in the (*E*)-series was considerably higher than that of the native hormone.

**Scheme 8:**
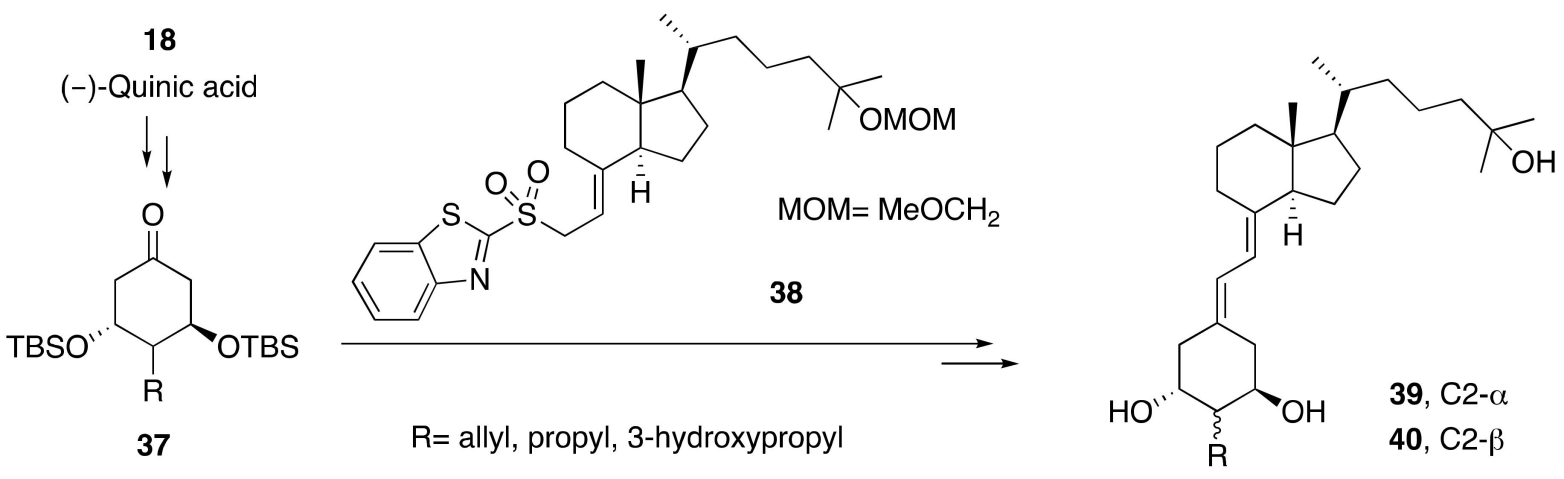
Preparation of C2-alkyl substituted 1α,25-(OH)_2_-19-*nor*-vitamin D_3_.

Novel C2-substituted analogs of 1α,25-(OH)_2_-19-*nor*-vitamin D_3_ were efficiently synthesized by Kittaka and co-workers [23,24]. The C2-alkyl A-ring precursors were prepared as (3*R*,5*R*)-4-alkyl-3,5-dihydroxycyclohexanones **37** (Scheme 8) from (–)-quinic acid through radical allylation at the C4 position of methyl (–)-quinate. A new type of the CD-ring coupling partner **38** with an elongated two-carbon unit was synthesized from 25-hydroxy Grundmann’s ketone. To construct a diene unit between the A-ring and the CD-ring, modified Julia olefination was used. The analog **39c** [C2α-(3-hydroxypropyl) derivative] showed an increase in both the vitamin D receptor (VDR) binding affinity and potency in the induction of HL-60 cell differentiation.

**Scheme 9:**
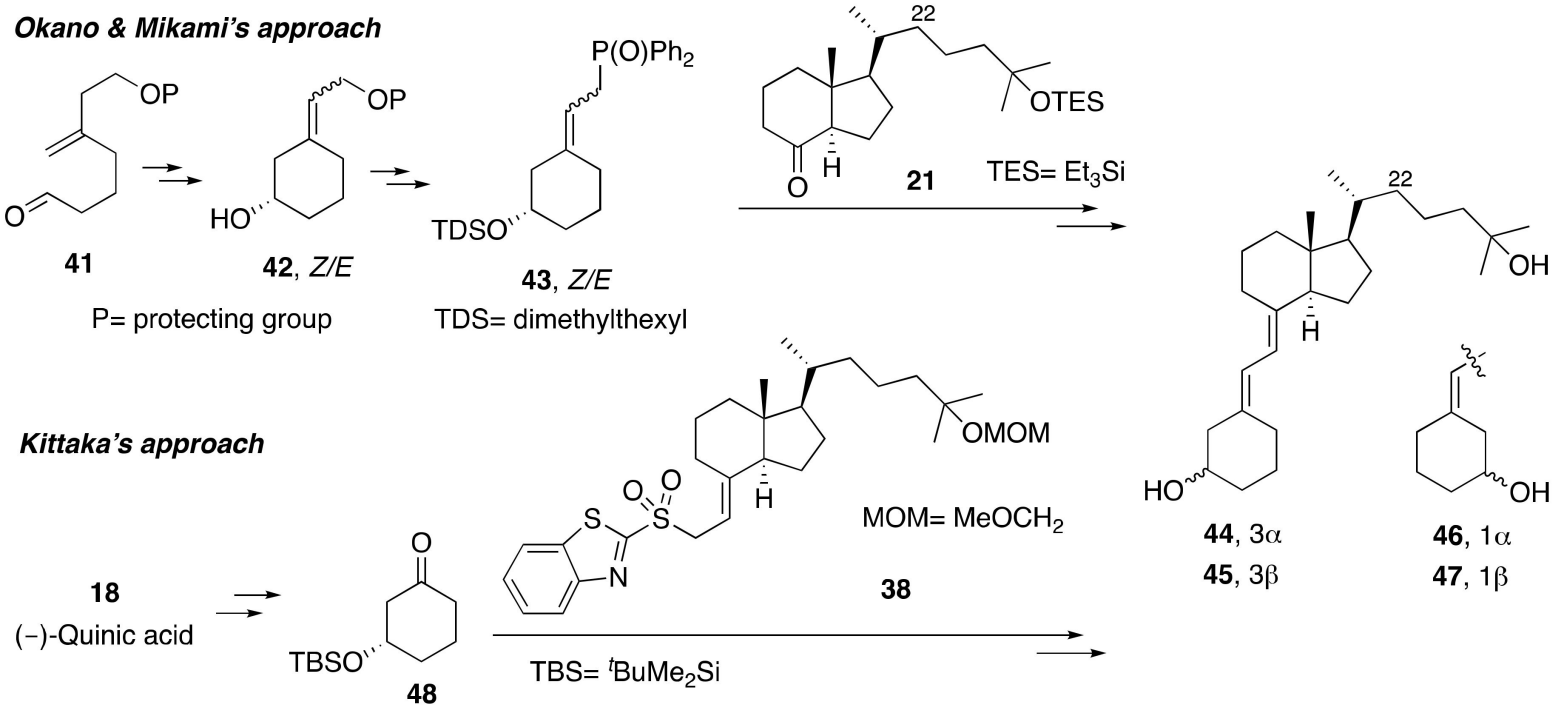
Preparation of singly dehydroxylated 1α,25-(OH)_2_-19-*nor*-vitamin D_3_ A-ring analogs.

A series of singly dehydroxylated 19-*nor*-1α,25-dihydroxyvitamin D_3_ A-ring analogs (**44**–**47**, Scheme 9) were synthesized by Okano, Mikami, and co-workers [25]. Starting from an aldehyde **41**, A-ring synthons **42** were prepared. The key feature in the syntheses is the stereospecific transformation of the (*E*)- and (*Z*)-ene cyclization products into the phosphine oxide **43**. Wittig–Horner coupling of the Grundmann’s ketone derivative **21** with the former synthon **43** led to the analogs **44**–**47**. These analogs are useful probes to assess the biological roles of hydroxyl groups at C1 and C3 independently.

An alternative approach was presented by Kittaka’s group [26] using a new Julia-type olefination. The A-ring precursor **48** (Scheme 9) was synthesized from quinic acid in an enantiomerically pure form. The key step was Julia-type olefination, which proceeded with high yields via the reaction of the ketone **48** with a sulfone **38** to form the protected 19-*nor*-vitamin D derivatives as a 1:1 diastereomeric mixture **45**:**46**.

**Scheme 10:**
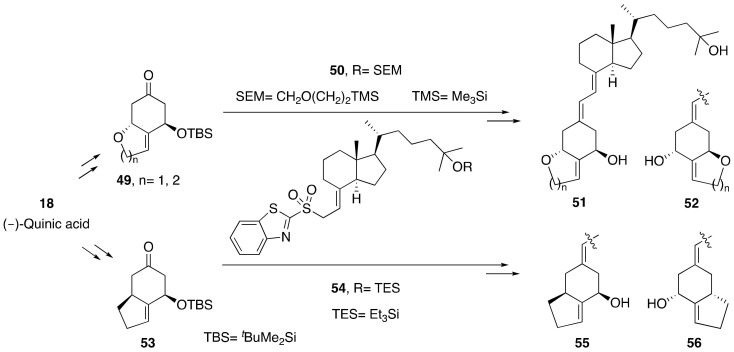
Synthesis of 19-*nor*-vitamin D_3_ analogs with a frozen A-ring conformation.

To establish the conformation of vitamin D compounds responsible for biological activity, a 1α,25-dihydroxy-19-*nor*-vitamin D analog **51** (Scheme 10) possessing a 1α-hydroxy group fixed in the axial orientation (β-chair form) was synthesized by DeLuca’s group [27]. The starting material to prepare the A-ring synthon was quinic acid, which was converted to the bicyclic ketones **49**. Julia coupling of the latter with a sulfone **50** produced the 19-*nor*-vitamin D analog **51b** (*n* = 2), which possessed an additional ring connecting the 3β-oxygen and C2, and the isomeric 3β-hydroxy compound **52b** (*n* = 2). Similar analogs **51a** and **52a** (*n* = 1), with even more rigid conformation, were also described [28]. The bicyclic fragment **49a** (*n* = 1), which was obtained using a ring-closing metathesis process, reacted with the sulfone **50** in Julia coupling conditions to yield derivatives **51a** and **52a**. Unexpectedly, these compounds exhibited a calcemic response, probably due to their metabolic conversion in living organisms. To verify this hypothesis, DeLuca and co-workers [29] focused their attention on preparing analogs in which the additional ring possessed only carbon units (**55** and **56**). To obtain the latter compounds, a similar procedure to that above was followed with Grundmann’s ketone **54** and the A-ring synthon **53** that comes from (−)-quinic acid in a multi-step synthesis.

**Scheme 11:**
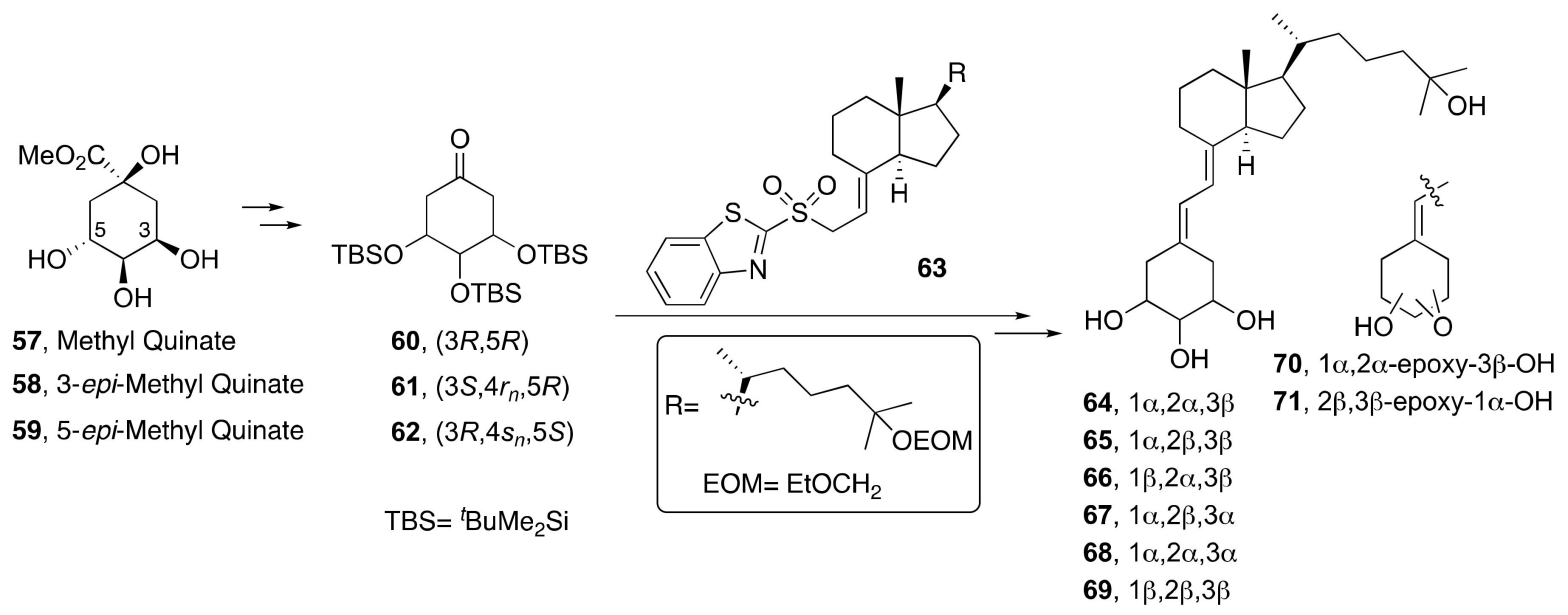
Preparation of trihydroxylated and epoxides of 1α,25-(OH)_2_-19-*nor*-vitamin D_3_ A-ring analogs.

Because of the importance of A-ring stereochemistry in the biological responses, we previously described [30] an improved method to synthesize of C2-hydroxy (**64**–**69**, Scheme 11) and C2,C3- or C1,C2-epoxi-substituted (**70**,**71**) 1α,25-dihydroxy-19-*nor*-vitamin D_3_ analogs with different configurations of the A-ring at C1, C2, and C3. A-Ring synthon precursors **60**–**62** were prepared from methyl quinate and its 3-*epi* and 5-*epi*-isomers. The CD-ring/side chain fragment **63** was derived from Grundmann’s ketone by oxidizing the 25-position and then protecting the hydroxyl group as ethoxymethyl ether (EOM). Modified Julia olefination between the ketones **60**–**62** and the sulfone **63** yielded the vitamin D analogs **64**–**71**.

**Scheme 12:**
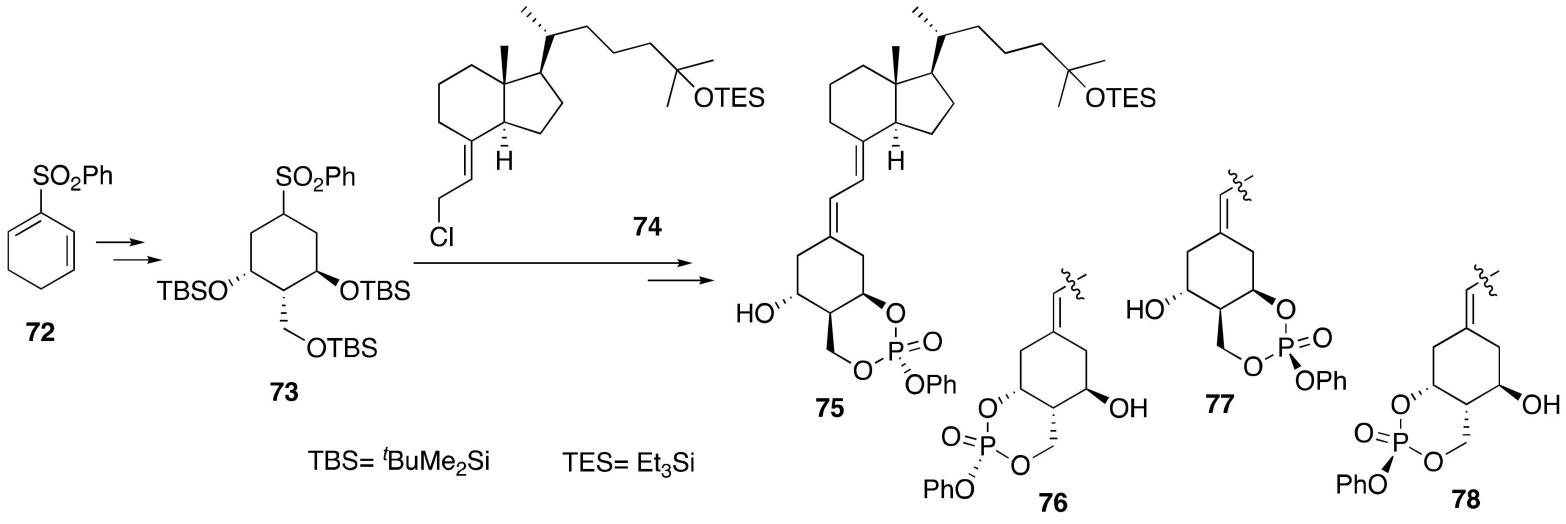
Preparation of C2 cyclic phosphate 1α,25-(OH)_2_-19-*nor*-vitamin D_3_ A-ring analogs.

It has been reported that the introduction of a phosphonate group to the upper side chain of vitamin D analogs reduces their calcemic effects. With the aim to test the effects on VDR binding affinity and hypercalcemic properties, phosphorus-bearing A-ring derivatives were designed. A highly convergent synthesis of four 19-*nor*-vitamin D_3_ cyclic phosphates (**75**–**78**, Scheme 12) was achieved [31] via a reaction of a sulfonate **73** with a CD-ring allyl chloride **74**. The A-ring synthon precursor **73** was synthesized from an achiral dienyl sulfone **72** as the preliminary starting material, which was prepared on a kilogram scale.

**Scheme 13:**
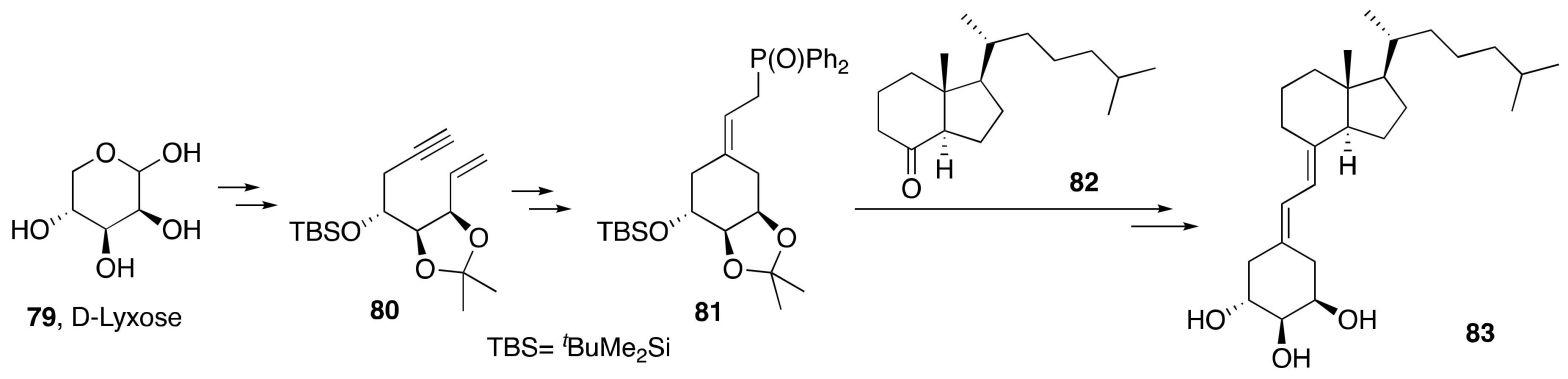
Preparation of trihydroxy 1α,2β-(OH)_2_-19-*nor*-vitamin D_3_.

Okamoto and co-workers [32] developed a synthetic method for preparing 2-hydroxy-type A-ring precursors **81** (Scheme 13) via a common intermediate **80** from d-lyxose (**79**). This involved a Ti(II)-mediated ene-yne cyclization and a Ru–carbene-catalyzed ene-yne metathesis followed by a Ni-catalyzed regioselective hydroboration of the 1,3-diene as the key-step reactions. The resulting A-ring moiety, containing 1,2,3-triol functionality, can be selectively silyl-protected to produce 1,3-di(silyloxy)-2-hydroxy products, which are useful for further manipulation at the C2-position.

**Scheme 14:**
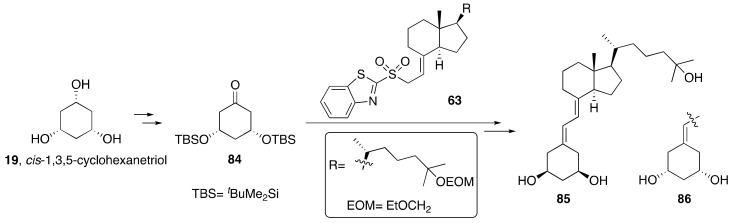
Preparation of 1α,25-(OH)_2_-19-*nor*-vitamin D_3_ A-ring C1- and C3-*epi* analogs.

Our laboratory recently reported [33] structurally simple but synthetically challenging A-ring epimers at C1 and C3 of the 1α,25-(OH)_2_-19-*nor*-vitamin D_3_ (Scheme 14). These 19-*nor* analogs had not been described previously, probably because the structure is difficult to elucidate. These analogs (1-*epi*- **86** and 3-*epi*-1α,25-(OH)_2_-19-*nor*-D_3_ **85**) were synthesized via a convergent synthesis starting from *cis,cis*-1,3,5-cyclohexanetriol (**19**), a commercially available compound, and the protected 25-hydroxy Grundmann’s ketone **63**. Modified Julia olefination between a ketone **84** and sulfone **63** gave rise to the target compounds **85** and **86**. An alternative synthesis using an orthogonally protected intermediate was necessary to unambiguously assign the corresponding structures to each diastereoisomer by NMR spectroscopy. The key step was the biocatalytic desymmetrization of the mono-TBDPS-protected derivative of *cis,cis*-1,3,5-cyclohexanetriol.

### 3.1. Synthesis of A-Ring- and Diene-Modified Analogs

**Scheme 15:**
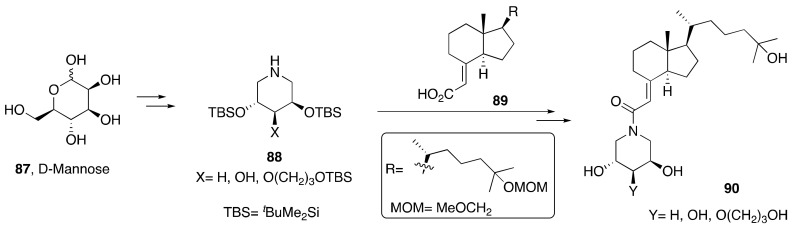
Preparation of 1α,25-(OH)_2_-19-*nor*-vitamin D_3_ C2-substituted amide A-ring analogs.

Takayama’s research group [34] focused on the modification of the A-ring and diene moieties in the 19-*nor* skeleton. Thus, they described the synthesis of new 1α,25-dihydroxy-19-*nor*-vitamin D_3_ analogs **90** (Scheme 15), which have an amide bond in the molecule instead of the diene. The A-ring moiety was substituted with a piperidine ring that has two hydroxyls at C1α and C3β with the appropriate stereochemistry. The A-ring synthons **88** were obtained from d-mannose (**87**), and the CD-ring carboxylic acid **89** was synthesized from Grundmann’s ketone. Subsequent condensation of **89** with the piperidine derivatives **88** provided the amides **90** in good yields after deprotection of the silyl groups. This strategy can be applied in combinatorial chemistry; therefore, these compounds would be applicable as useful tools in the development of new drugs.

**Scheme 16:**
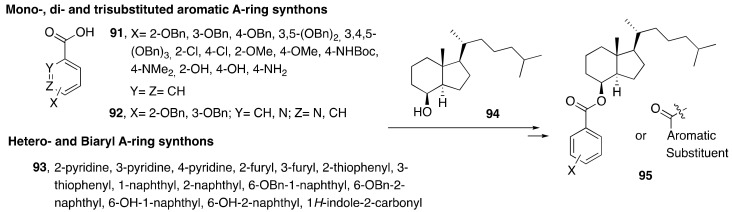
Preparation of 1α,25-(OH)_2_-19-*nor*-vitamin D_3_ analogs with an aromatic A-ring.

Hadden and co-workers [35] conducted a structure−activity relationship study using a series of vitamin D_3_-based analogs with aromatic A-ring mimics that resulted in potent, selective Hedgehog pathway inhibition. Thus, they reported the synthesis of aromatic A-ring modified derivatives containing single or multiple substitutions in the aromatic A-ring (**91** and **92**, Scheme 16) or a heteroaryl or biaryl moiety (**93**). As precursors of A-ring synthons **91**–**93**, commercially available aromatic acids and esters were purchased and either coupled directly to **94** or coupled following standard protection/hydrolysis strategies.

### 3.2. Synthesis of A-Ring-, D-Ring-, and Side-Chain-Modified Analogs

**Scheme 17:**
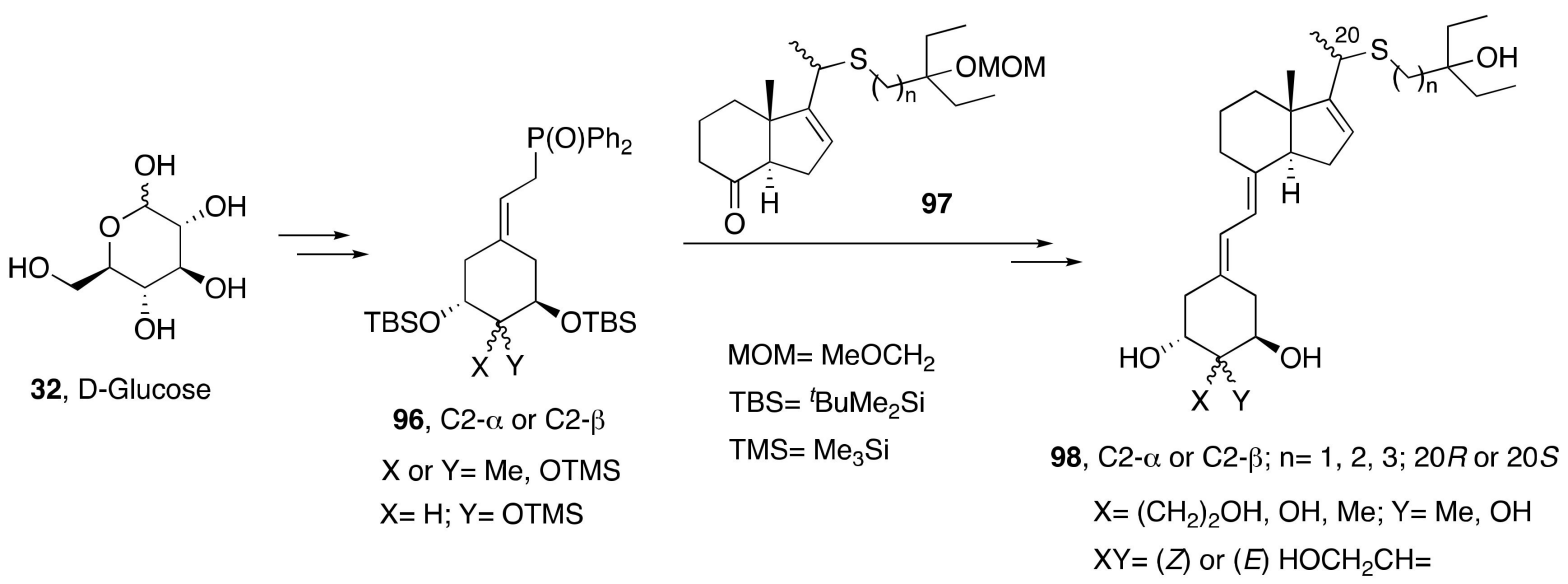
Synthesis of 1α,25-(OH)_2_-19-*nor*-vitamin D_3_ analogs with A-ring, D-ring, and side-chain modifications.

To further investigate the effects of the A-ring modification of 16-ene-22-thia-26,27-dimethyl-19-*nor*-vitamin D_3_ analogs **98** (X = Y = H; *n* = 2, 3; Scheme 17) on the biological activity profile, novel 22-thia-19-*nor*-vitamin D analogs bearing a hydroxyethoxy-, hydroxyethylidene-, or methyl group at C2 in combination with 20*S*- and 20*R*-isomers were prepared and tested by Ito and co-workers [36]. For the synthesis of 2-substituted-16-ene-22-thia-19-*nor*-vitamin D analogs **98**, they employed a Wittig–Horner coupling of the appropriately substituted A-ring phosphine oxides **96**, prepared from d-glucose, with the 25-hydroxy Grundmann’s ketone derivatives **97**.

### 3.3. Synthesis of A-Ring-, CD-Ring-, and Side-Chain-Modified Analogs

**Scheme 18:**
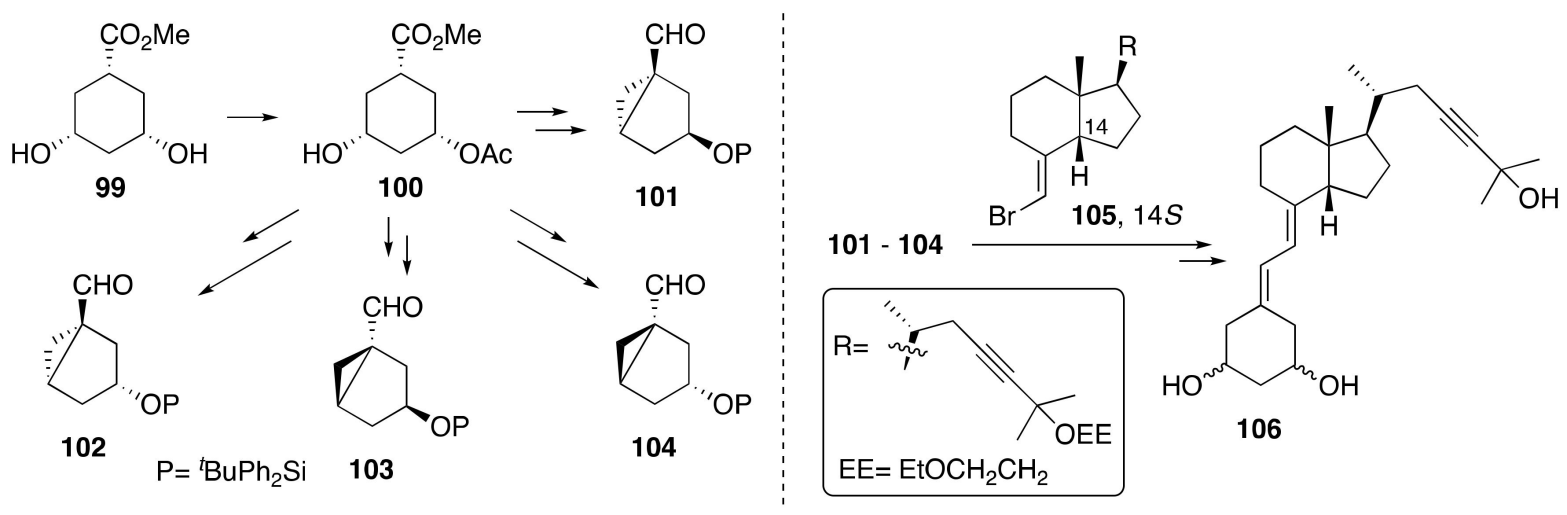
Synthesis of 1α,25-(OH)_2_-19-*nor*-vitamin D_3_ analogs with A-ring, CD-ring, and side chain modifications.

Vandewalle’s group [37] reported a practical method to synthesize of (2*S*,3a*S*,4a*S*)-2-*tert*-butyldimethylsilyloxybicyclo[3.1.0]hexane-3a-carbaldehyde (**101**, Scheme 18) and diastereoisomers (**102**–**104**), starting from all-*cis* methyl 3,5-dihydroxy-1-cyclohexanecarboxylate (**99**). The key step was the asymmetrization of **99** by an enzyme-catalyzed transesterification, which afforded crystalline enantiopure **100** in high yield. Several chemical transformations led to cyclovitamin A-ring precursors **101**–**104**, which were coupled with appropriate *cis*-hydrindane **105** to produce the 14-*epi*-19-*nor* vitamin D_3_ analogs **106**. This method is applicable to the large-scale production.

### 3.4. Synthesis of A-Ring- and Side-Chain-Modified Analogs

The first analogs of this class were reported by Mikami’s group [25]. They consist of several singly dehydroxylated 22-oxa-(1α),25-(OH)_2_-19-*nor*-vitamin D_3_ derivatives with similar structures to those of compounds **44**–**47** (Scheme 9) in which the CH_2_ in the 22-position was replaced by an oxygen atom. Their syntheses were similar to that described in Scheme 9 but with the corresponding 22-oxa-CD-ring/side chain synthon of **21**. These analogs were synthesized since little information was available concerning the structural motifs of the 1α,25-(OH)_2_-D_3_ hormone responsible for the modulation of differentiation and apoptosis.

**Scheme 19:**
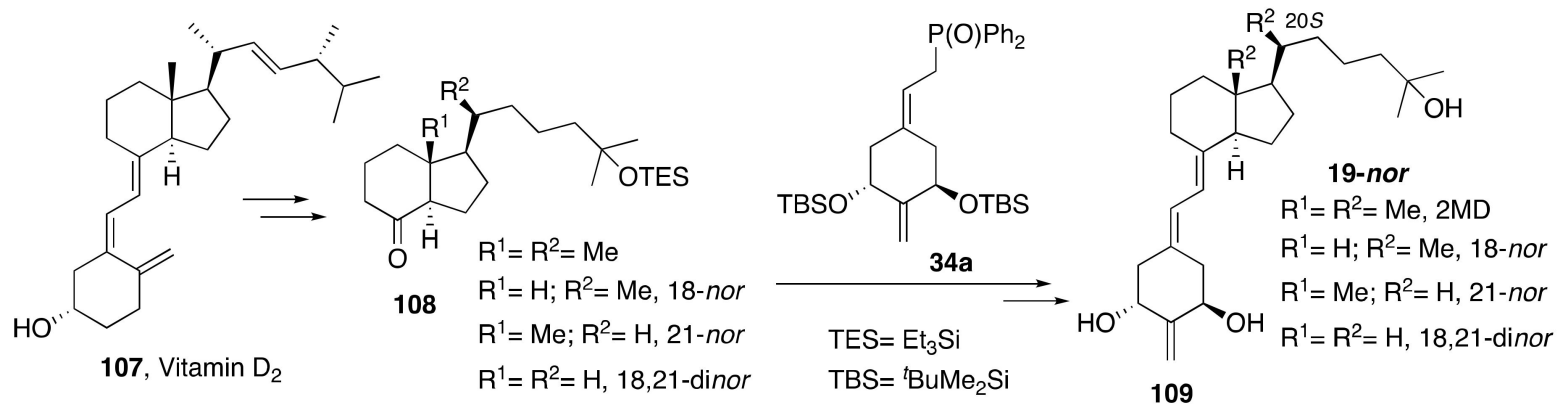
Synthesis of 2MD *nor* and di*nor* derivatives of 1α,25-(OH)_2_-19-*nor*-vitamin D_3_.

Another family of compounds was described by DeLuca’s group [20]. Their structure is similar to that of product **35a** (R = H, Scheme 7) but with the unnatural 20*S* configuration. The latter compound, named 2MD ((20*S*)-1α,25-dihydroxy-2-methylene-19-*nor*-vitamin D_3_), showed a unique ability to induce bone formation and has proven anabolic for bone in in vivo studies, but it has a tendency to cause hypercalcemia because of its bone calcium mobilizing activity. For this reason, DeLuca and co-workers [38] prepared several derivatives of 2MD **109** (Scheme 19). Thus, the 18-*nor*, 21-*nor,* and 18,21-di*nor* analogs of 2MD **109** were prepared by convergent syntheses. The known phosphine oxide **34a**, obtained from quinic acid, was coupled by the Wittig–Horner process with the corresponding CD-ring/side chain fragments **108**, obtained by a multi-step procedure from commercial vitamin D_2_ (**107**).

**Scheme 20:**
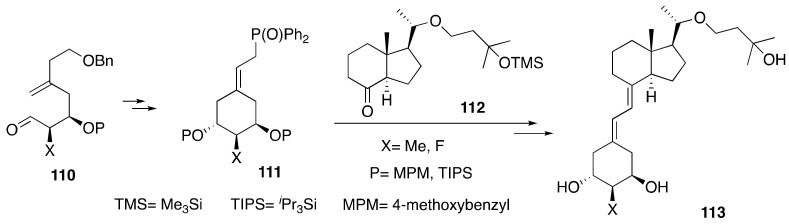
Synthesis of C2-methyl and C2-fluoro 22-oxa-1α,25-(OH)_2_-19-*nor*-vitamin D_3_ derivatives.

Mikami and co-workers showed the synthesis of hybrid analogs of 2-methyl- (**113a**) [39] and 2-fluoro-22-oxa-1α,25-(OH)_2_-19-*nor*-D_3_ (**113b**) [40] (Scheme 20). One of the reasons that they synthesized fluorinated A-ring analogs was to investigate the VDR-binding conformation of the A-rings on the basis of ^19^F NMR analysis. The A-rings **111** were synthesized via asymmetric catalytic carbonyl-ene cyclization from aldehydes **110**. Subsequently, a fluorine A-ring synthon **111** was synthesized via the highly regio- and stereoselective epoxide-opening 2α-fluorination and catalytic asymmetric carbonyl-ene cyclization [41]. Lythgoe coupling of the 22-oxa-CD-ring/side chain **112** with the Wittig reagents **111** led, via desilylation, to C2-methyl and C2-fluoro-1α,25-(OH)_2_-19-*nor*-D_3_ analogs **113**.

**Scheme 21:**
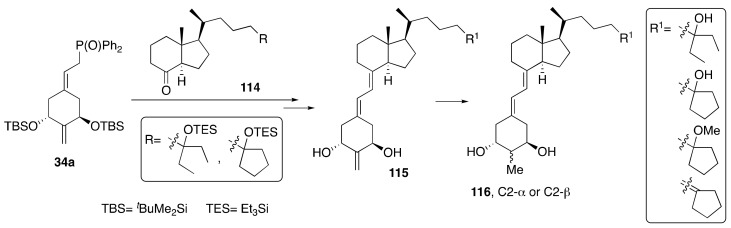
Synthesis 2-substituted side-chain-modified (20*S*)-1α,25-(OH)_2_-19-*nor*-vitamin D_3_.

In light of previously reported data, there was an interest in preparing vitamin D derivatives with more potent calciotropic activity than that of the natural hormone because of their potential use in the treatment of osteoporosis. DeLuca and co-workers [21] became interested in the exploration of the effect of the side chain structure on the activity of 2-methylene and 2-methyl substituted analogs. Thus, they described multiple modified analogs similar to **35** and **36** shown in Scheme 7, the only difference is in the stereochemistry at C20. Instead of just the natural configuration (C20*R*), they also obtained epimers, that is, C20*S*. They also prepared [42] 2-substituted (20*S*)-1α,25-dihydroxy-19-*nor* vitamin D_3_ analogs **115** and **116** (Scheme 21) with elongated side chains by Wittig–Horner coupling of an A-ring phosphine oxide **34a** with the corresponding protected (20*S*)-25-hydroxy Grundmann’s ketones **114**.

**Scheme 22:**
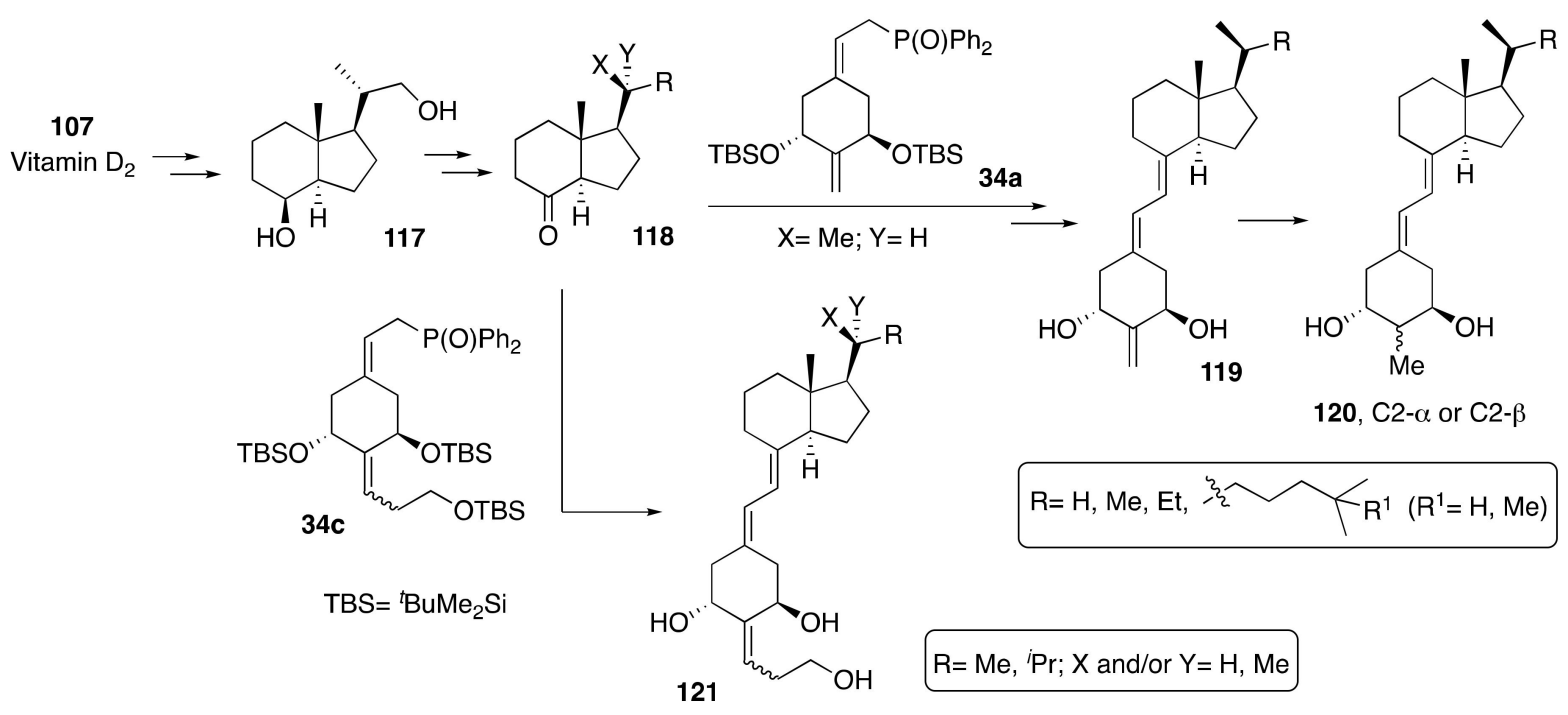
Synthesis of C2-substituted 1α-OH-19-*nor*-vitamin D_3_ with shortened side chains.

Using their procedure, DeLuca’s group synthesized C2-substituted 1α-OH-19-*nor*-vitamin D_3_ with C20*S* alkyl side chains (Scheme 22). They prepared an abbreviated [43], 25-methyl substituted, and normal side chain containing no hydroxyl group [44]. The key step in the synthesis was the Wittig–Horner olefination of Grundmann’s ketones **118** possessing different 17β-alkyl substituents, with the phosphine oxide **34a** prepared from (–)-quinic acid. The corresponding CD fragment **118** was obtained from the known Inhoffen–Lythgoe diol **117**, which is easily obtained from vitamin D_2_ (**107**). The coupling of ketones **118** with the anion generated from **34a**, followed by hydroxyl deprotection, generated the corresponding 2-methylene-19-*nor*-vitamins **119** and, after homogeneous catalytic hydrogenation, provided 2-α/β-methyl-19-*nor*-vitamins **120**. On the other hand, 19-*nor*-vitamins **121** possessing a 3′-hydroxypropylidene fragment attached to C2 and shortened 17β-alkyl chains have also been reported [45]. The hydrindanones **118** were subjected to a reaction with the phosphine oxide **34c**, and the vitamin D compounds **121** were obtained after hydroxyl deprotection.

**Scheme 23:**
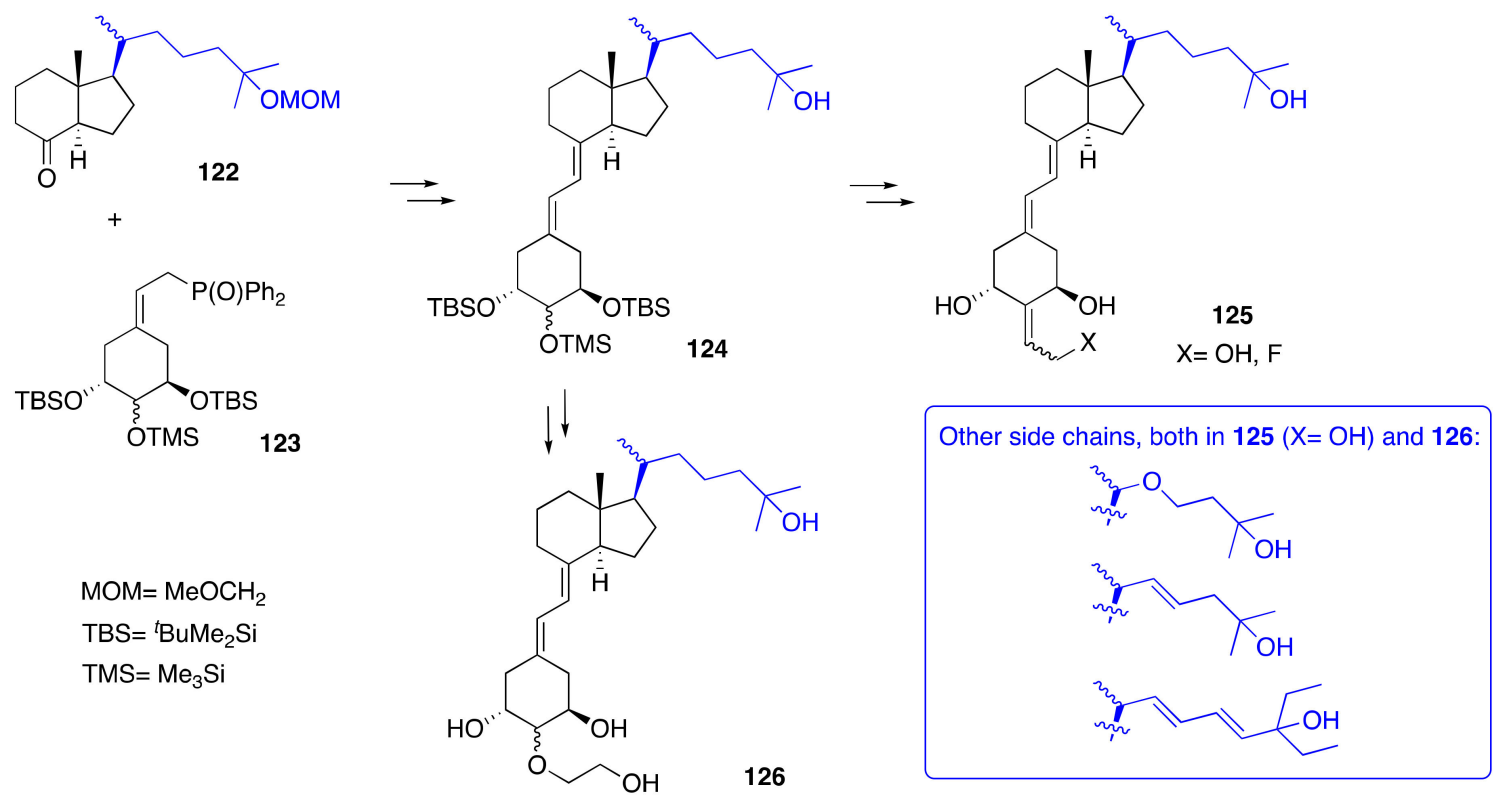
Synthesis of 2-hydroxy- and 2-fluoroethylidene side-chain-modified analogs of 1α,25-(OH)_2_-19-*nor*-vitamin D_3_.

To further examine the effects on binding affinity to VDR as well as the transcriptional activity of 2-substituted 19-*nor*-vitamin D analogs, Shimizu and coworkers [46,47,48] synthesized new 2-substituted and side-chain-modified 19-*nor*-1α,25-(OH)_2_-D_3_ derivatives. 25-Hydroxy-protected Grundmann’s ketones **122** (Scheme 23) were treated with (3*R*,5*R*)-3,4,5-trihydroxy A-ring phosphine oxide derivatives **123**, which were synthesized enantioselectively from d-glucose, to generate vitamin D intermediates **124**. The latter compounds were unprotected at C2-OTMS and oxidized to ketones and, after several transformations, yielded 2-hydroxyethylidene derivatives **125** (X = OH) or 2-fluoroethylidene analogs **125** (X = F) as a 1:1 mixture of *E-* and *Z*-isomers. OTMS-unprotected derivatives of **124** were allowed to react with 2-bromoethanol TBS ether, and then all protecting groups were removed, yielding the analogs **126**. Both families of compounds **125** and **126** were also obtained with other side chains starting from the appropriately modified CD-ring/side chain **122**.

**Scheme 24:**
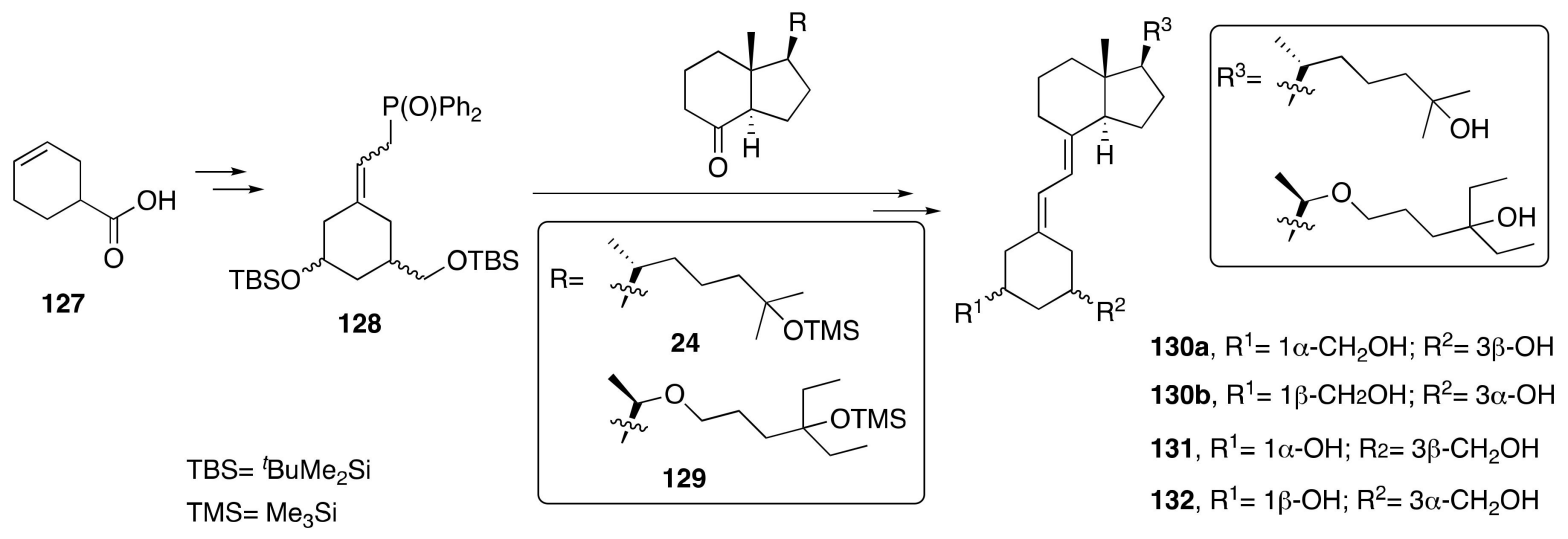
Synthesis of hydroxymethyl and side chain derivatives of 1α,25-(OH)_2_-19-*nor*-vitamin D_3_.

Posner and co-workers [49] prepared a series 19-*nor* analogs **130**–**132** (Scheme 24) of the hormone calcitriol containing a hydroxymethyl group either at the 1-position or at the 3-position. They wanted to determine whether the 19-*nor*-D derivatives behaved like natural vitamin D but with even less of a calcemic effect. The synthesis of the A-ring synthons **128** started from readily available 3-cyclohexene-1-carboxylic acid (±)-**127**. Vitamin D 19-*nor* analogs **130**–**132** were prepared through Horner–Wadsworth–Emmons coupling of the corresponding CD-ring ketones **24** or **129** and A-ring phosphine oxides **128**. 

**Scheme 25:**
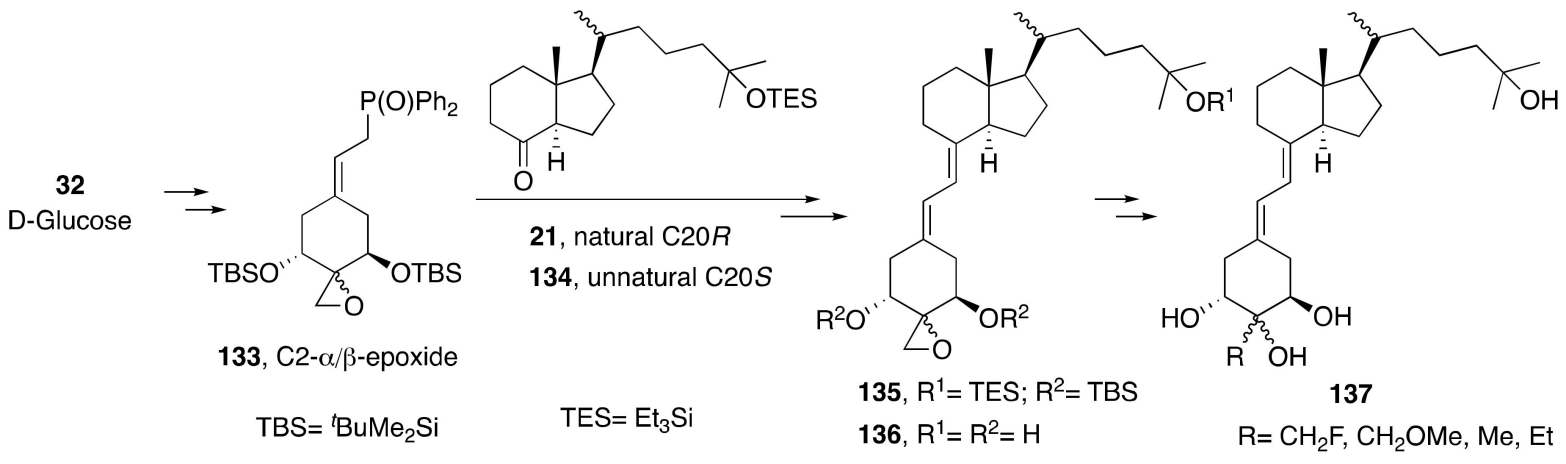
Synthesis of 2,2-disubstituted-(20*R*)- and (20*S*)-1α,25-(OH)_2_-19-*nor*-vitamin D_3_.

To examine the effect of 2,2-disubstitution on the biological activities of 19-*nor*-vitamin D derivatives, Shimizu’s group [50] described the synthesis of novel 2,2-disubstituted-(20*R*)- and (20*S*)-19-*nor*-vitamin D_3_ analogs **136** and **137** (Scheme 25). For the synthesis of the target 2,2-disubstituted compounds **136**–**137**, they used a Wittig–Horner coupling approach involving the A-ring phosphine oxide **133** with 25-hydroxy Grundmann’s ketones possessing a natural 20*R*-**21** and an unnatural 20*S*-**134** configuration. Deprotection of compound **135** led to the analogs **136** and, from nucleophilic ring-opening reactions, gave rise to the derivatives **137**.

DeLuca’s research group [22] described side-chain- and A-ring-modified analogs similar to compounds **35** with the unnatural C20*S* configuration using the same approach shown in Scheme 7.

**Scheme 26:**
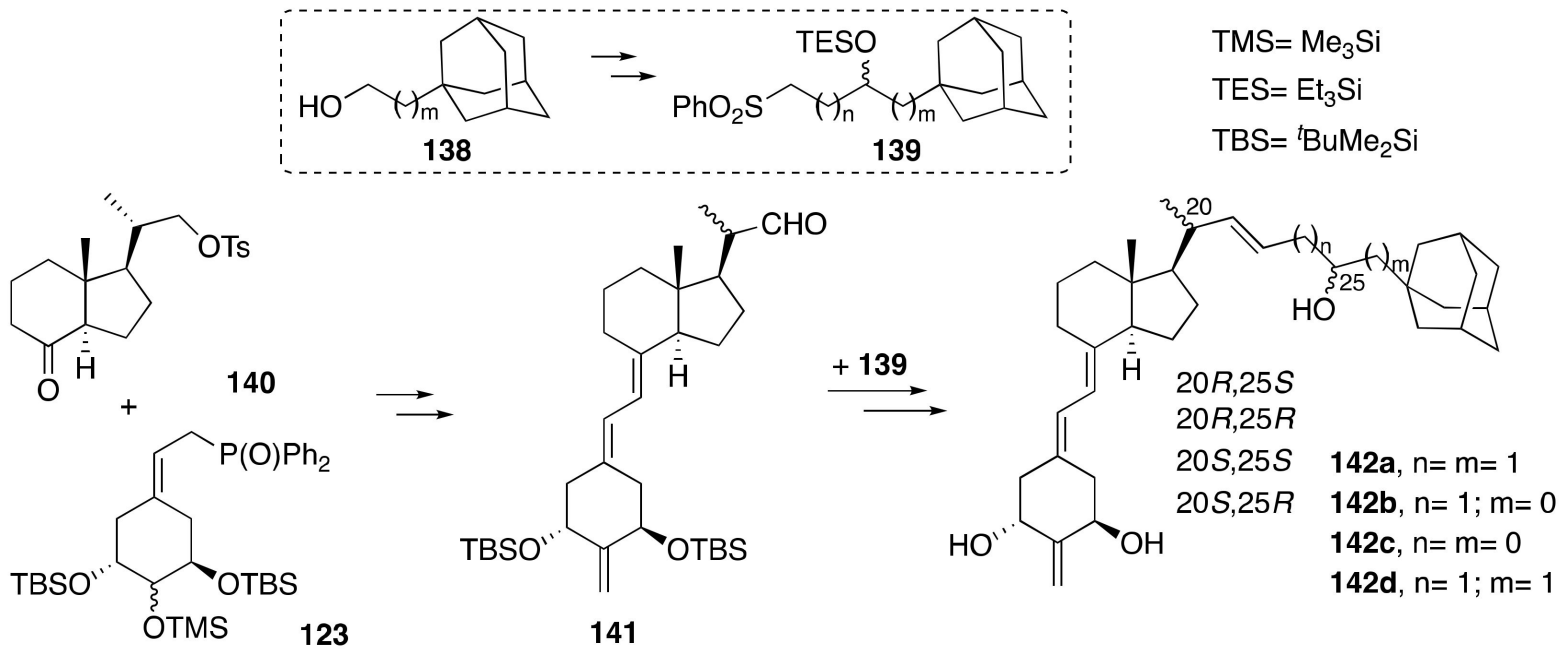
Synthesis of adamantyl derivatives of 1α,25-(OH)_2_-19-*nor*-vitamin D_3_.

Yamada and co-workers [51] synthesized four new vitamin D derivatives **142a** (Scheme 26), diastereomers at C20 and C25 of 26-adamantyl-1,25-dihydroxy-2-methylene-22,23-didehydro-19,27-dinorvitamin D_3_, which have a bulky and rigid adamantane ring system at the side chain terminus. Their synthesis was designed to fit into three parts: A side chain fragment with an adamantyl group at the terminal, a 19-*nor* A ring synthon, and a CD ring plus C22 side chain moiety. The side chain **139** was synthesized from adamantyl ethanol **138a** (*m* = 1). C22-Tosylate **140**, which was obtained from vitamin D_2_, was combined with 19-*nor*-A-ring phosphine oxides **123** to yield **141a** after several steps, and then both C22-aldehydes **141a** were coupled with the side chain sulfone **139a** and, after general functional group transformations, yielded the target vitamin D compounds **142a**. Several modifications of the length of the side chain were also reported [52] to generate the analogs **142b**-**142d**. These compounds have been shown to have a series of partial VDR agonistic/antagonistic activities. The bulky adamantyl side chain produces structural changes that destabilize the active protein conformation and reduce its contribution to equilibrium among the active and inactive conformations.

**Scheme 27:**
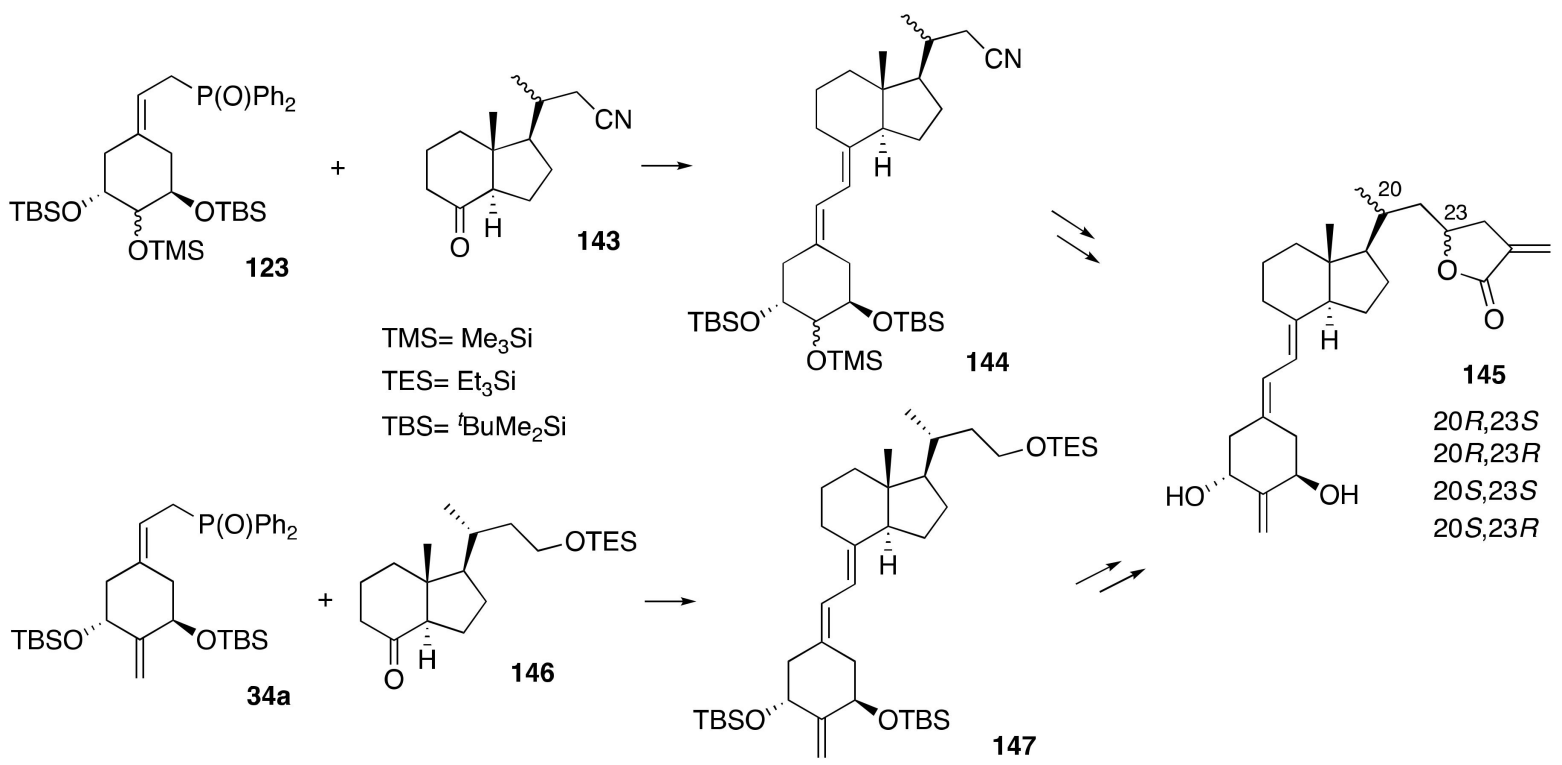
Synthesis of 25-methylene-26,23-lactone-2-methylene-1α-OH-19-*nor*-vitamin D_3_ analogs.

To investigate the molecular mechanism of VDR antagonists without a structurally bulky group interfering with helix 12 of the ligand-binding domain of the VDR, Yamada and Yamomoto’s group reported [53] the synthesis of four diastereomers at C20 and C23 of 1α-hydroxy-19-*nor*-vitamin D_3_ 25-methylene-26,23-lactone bearing a 2MD-type A-ring (**145**, Scheme 27). The Wittig–Horner reaction of Grundmann’s ketone derivatives **143** with A-ring phosphine oxides **123** derived from (−)-quinic acid resulted in the 23-cyanides **144**. Then, the procedure continued with 2-methylenation and subsequent methylene lactonization to yield the lactone analogs **145**. Furthermore, in an effort to explore the pharmacologically important C2-methylene derivatives of 19-*nor*-D_3_, DeLuca’s group [54] proposed another alternative pathway to synthesize compounds **145a**,**b** based on the same coupling approach of the Grundmann-type ketone **146** with a phosphine oxide **34a** to obtain the coupled product **147**, which, after several modifications, allowed them to isolate the analogs **145a**,**b.**

**Scheme 28:**
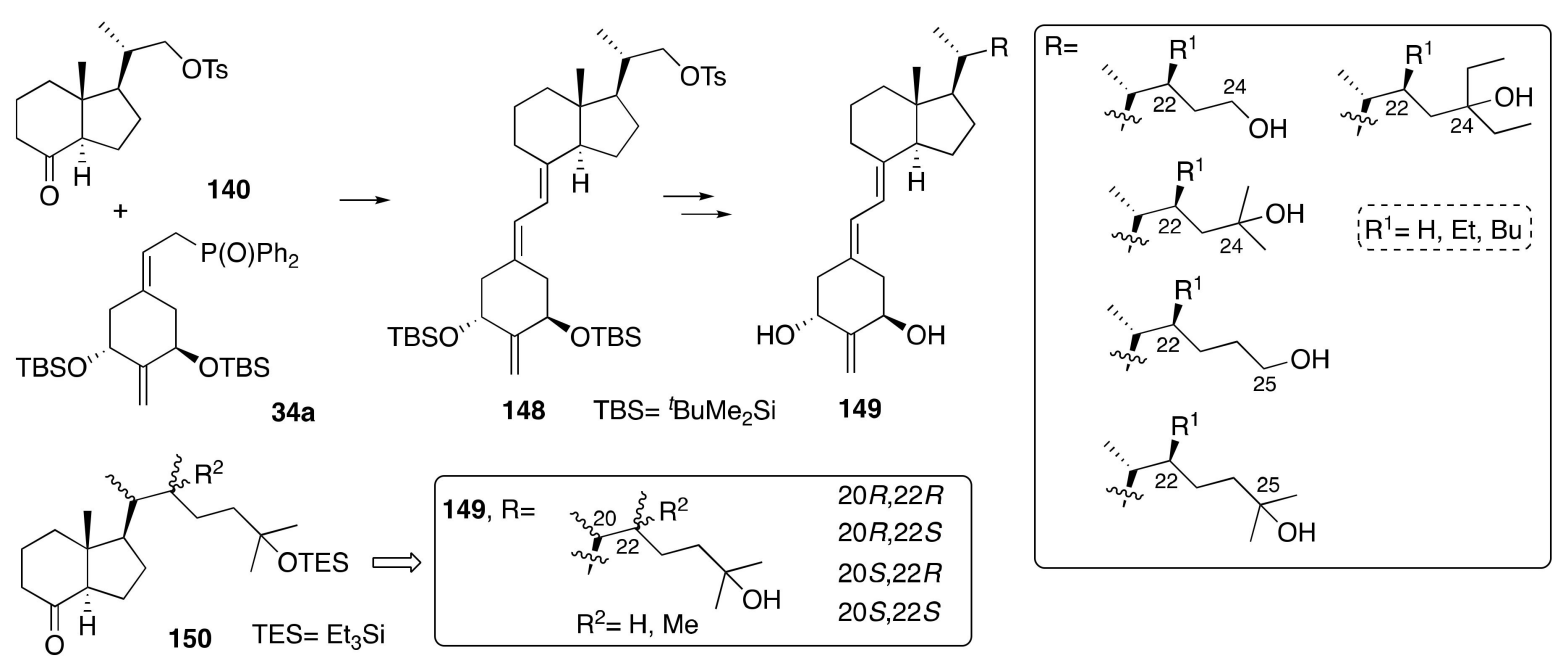
Synthesis of side chain 2-methylene-1α,25-OH-19-*nor*-vitamin D_3_ analogs.

A series of vitamin D_3_ analogs with or without a 22-alkyl substituent were synthesized by Yamamoto and co-workers [55] in order to evaluate their biological potency. 1,24- and 1,25-Dihydroxyvitamin D_3_ 22-alkyl derivatives **149** (Scheme 28) were synthesized from the corresponding tosylate **148**, which is a convenient synthetic intermediate for various vitamin D side-chain analogs, prepared from the coupling of the A-ring synthon **34a** and CD-ring moiety **140**. Later, the same group [56] synthesized analogs **149** with a more hydrophobic substituent at the 24-position to determine whether it could enhance VDR activation. Thus, they introduced a diethyl substituent instead of a dimethyl at C24. In addition, DeLuca’s group [57] prepared six new analogs of 1α,25-dihydroxy-19-*nor*-vitamin D_3_ **149** by a convergent synthesis and applied the Wittig−Horner reaction as a key step using the CD-ring/side chain derivative **150**. The objective was to test all possible conformers resulting from C20 epimerization and the introduction of a methyl group at the two available positions on C22. They also used the attachment of two methyl groups on C22 to help elucidate the biological impact of the double substitution of this side chain carbon.

**Scheme 29:**
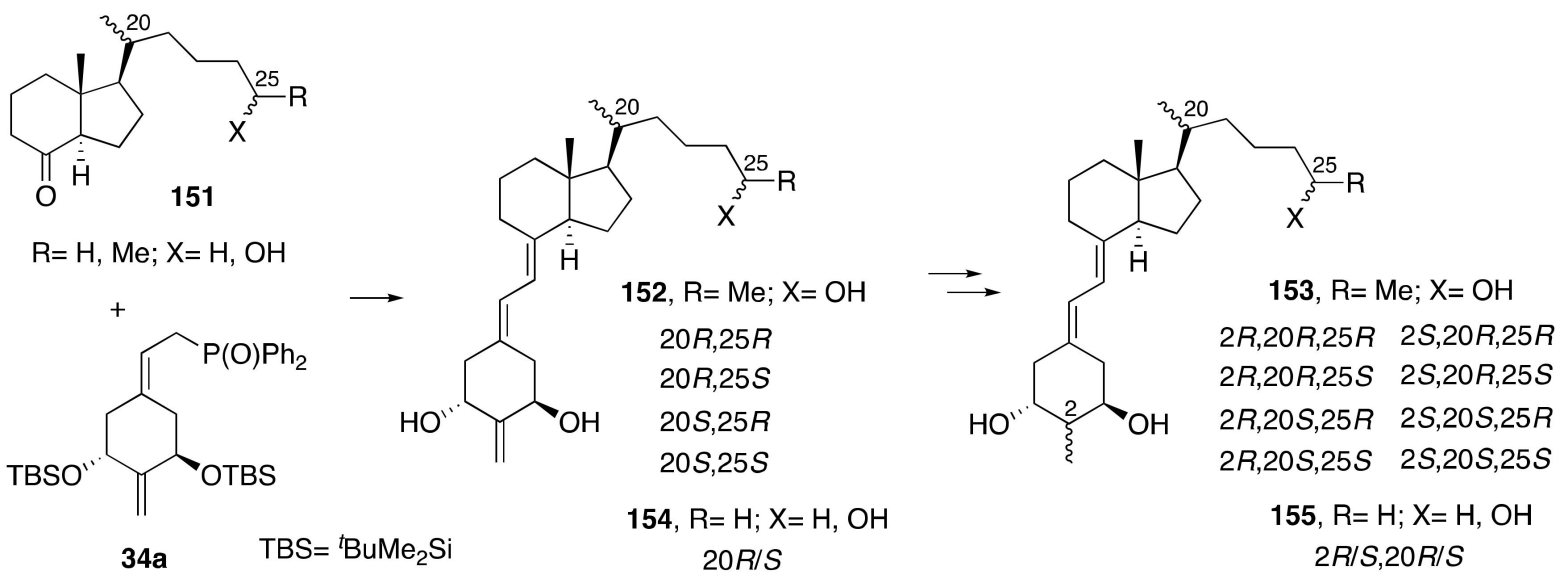
Synthesis of C25-(di)*nor* (di)methyl 2-substituted-1α,25-OH-19-*nor*-vitamin D_3_ analogs.

With the aim to test the influence of removing one [58] or both [59] methyl groups located at C25 on the biological in vitro and in vivo activity, DeLuca’s research group prepared 24 new analogs of 19-*nor*-1α,25-dihydroxyvitamin D_3_ (**152**–**155**, Scheme 29). To produce these compounds, a convergent approach via a Wittig–Horner reaction between Grundmann’s ketone derivatives **151**, either possessing fixed configurations of the hydroxyl group at C25 (X = OH) or lacking it (X = H), and the A-ring synthon **34a**. These syntheses led to the vitamin D derivatives **152** and **154** after deprotection of the silyl groups. The homogeneous catalytic hydrogenation of the 2-methylene moiety in **152** and **154** provided an equimolar mixture of 2-methyl-19-*nor*-vitamins **153** and **155**, which were easily separated by HPLC.

**Scheme 30:**
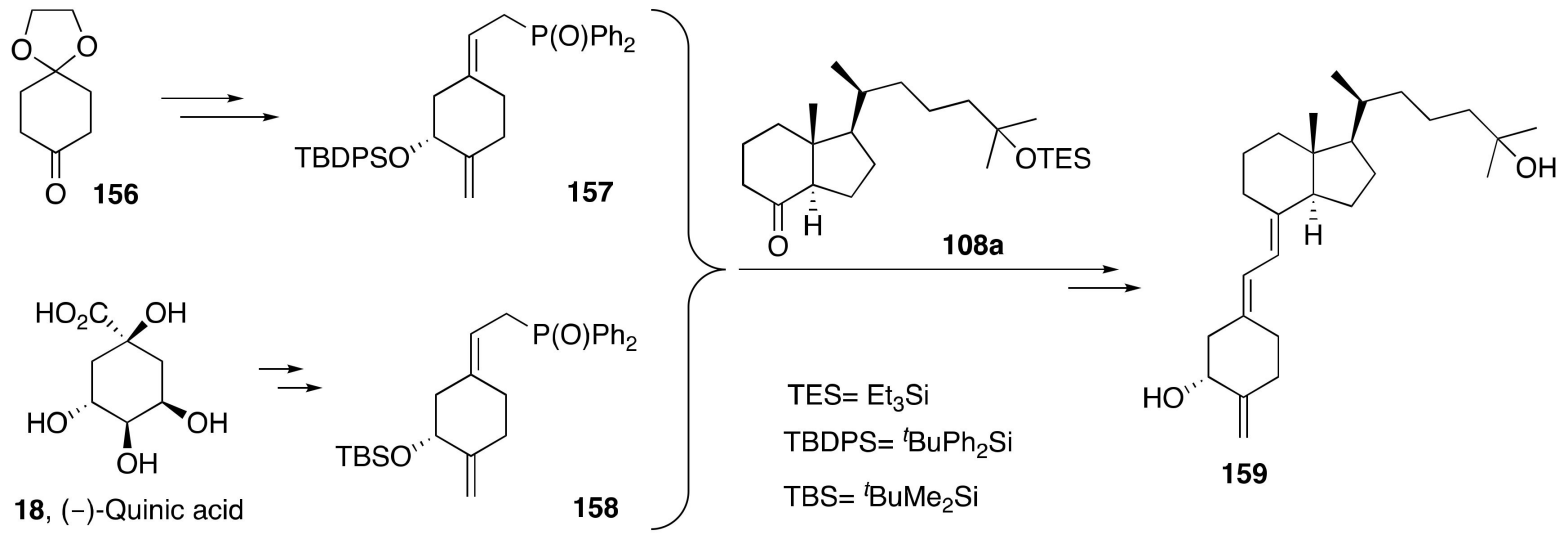
Synthesis of (20*S*)-25-hydroxy-2-methylene-19-*nor*-vitamin D_3_.

Compound (20*S*)-1α,25-dihydroxy-2-methylene-19-*nor*-vitamin D_3_, known as 2MD (2MD was discontinued from the phase II studies in 2011 since it lacked efficacy in increasing bone mineral density in osteopenic postmenopausal women, which is in contrast to its effect in the ovariectomized rats. The discrepancy could be due to the differences in bone metabolism in rats and humans.), significantly enhances calcemic activity and can be used for the treatment of osteoporosis. Therefore, it was of interest to also prepare its 1-desoxy analog **159** (Scheme 30). DeLuca’s group [60] reported that the synthesis of **159** by Wittig–Horner coupling of the known protected (20*S*)-25-hydroxy Grundmann’s ketone **108a** and the phosphine oxides **157** and **158**, which differ in their hydroxyl protection, provided the target 1-desoxy-2MD (**159**) after removal of the silyl protecting groups. Starting from commercially available compounds [1,4-cyclohexanedione monoethylene acetal **156** and (−)-quinic acid **18**)], two different routes were designed that led to both A-ring synthon precursors.

**Scheme 31:**
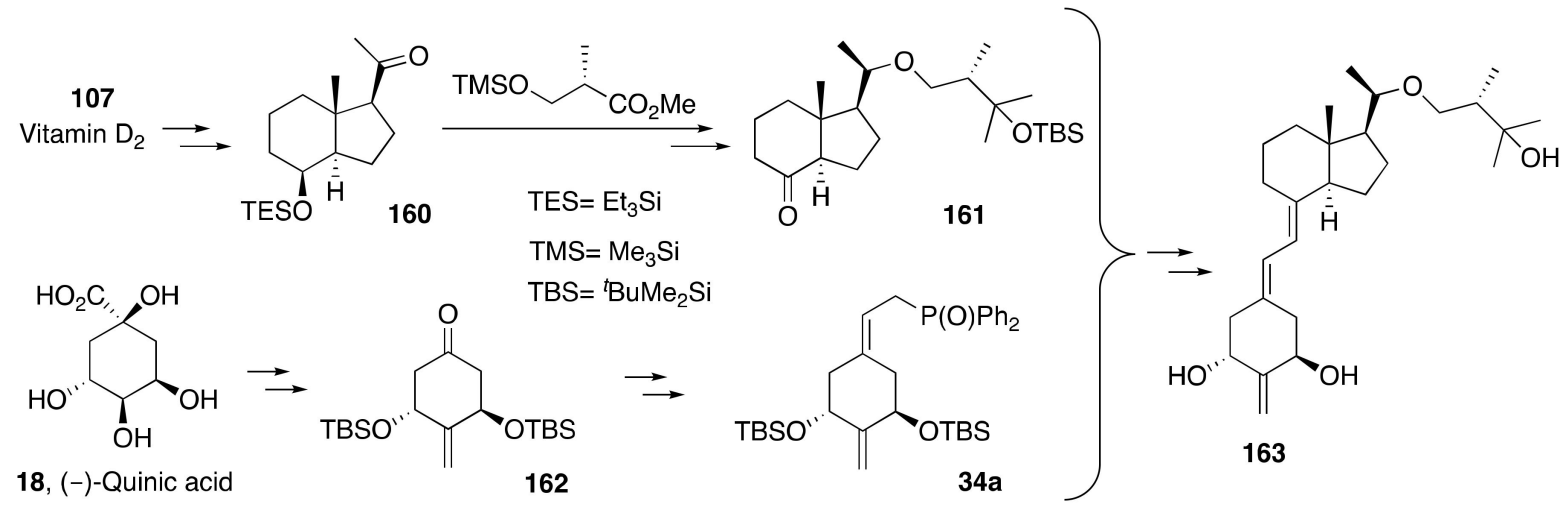
Synthesis of (20*S*)-24*R*-methyl-2-methylene-22-oxa-1α,25-dihydroxy-19-*nor*-vitamin D_3_.

A novel vitamin D receptor agonist **163** (Scheme 31) was synthesized by Wu-Wong and co-workers [61]. Preparation of A-ring diphenylphosphine oxide **34a** was obtained from a ketone **162**, which is easily available from commercial (−)-quinic acid. The CD-ring/side chain moiety **161** was prepared from the ketone **160**, which was ultimately obtained from vitamin D_2_. Wittig–Horner coupling of **34a** with the protected 25-hydroxy Grundmann’s ketone **161** generated the target compound **163** after the deprotection of TBS-protecting groups.

## 4. Synthesis of Diene-Modified 1α,25-(OH)_2_-19-*nor*-Vitamin D

**Scheme 32:**
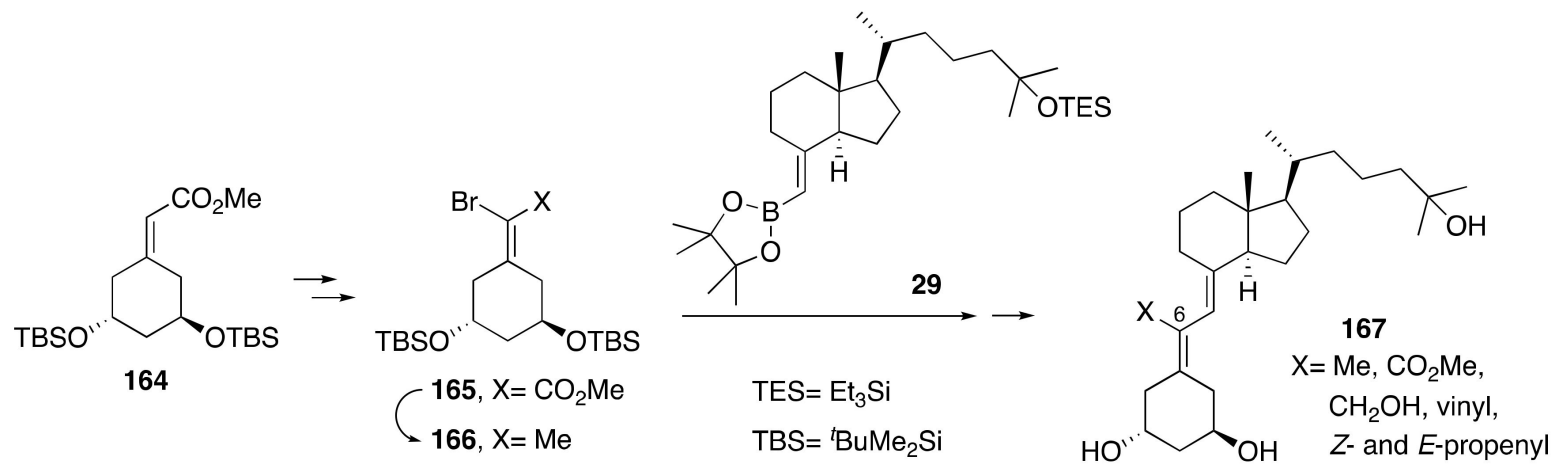
Synthesis of 6-substituted 1α,25-dihydroxy-19-*nor*-vitamin D_3_.

DeLuca’s group [62] previously reported the synthesis of a C6-methyl-substituted derivative of 1α,25-(OH)_2_-D_3_. This analog rearranges easily to its previtamin form and binds VDR very effectively. In order to test this modification in the vitamin form, a series of 6-substituted analogs of 1α,25-dihydroxy-19-*nor*-vitamin D_3_ were prepared, which are unable to undergo a conversion to their respective previtamin forms. The syntheses of analogs **167** (Scheme 32) bearing different substituents at C6 were accomplished by Suzuki–Miyaura cross-coupling reactions of a bicyclic organoboron derivative **29** with the respective alkenyl halides **165** or **166**, synthesized from precursor **164**.

## 5. Synthesis of CD-Ring-Modified 1α,25-(OH)_2_-19-*nor*-Vitamin D

### 5.1. Synthesis of C-Ring- and Side-Chain-Modified Analogs

**Scheme 33:**
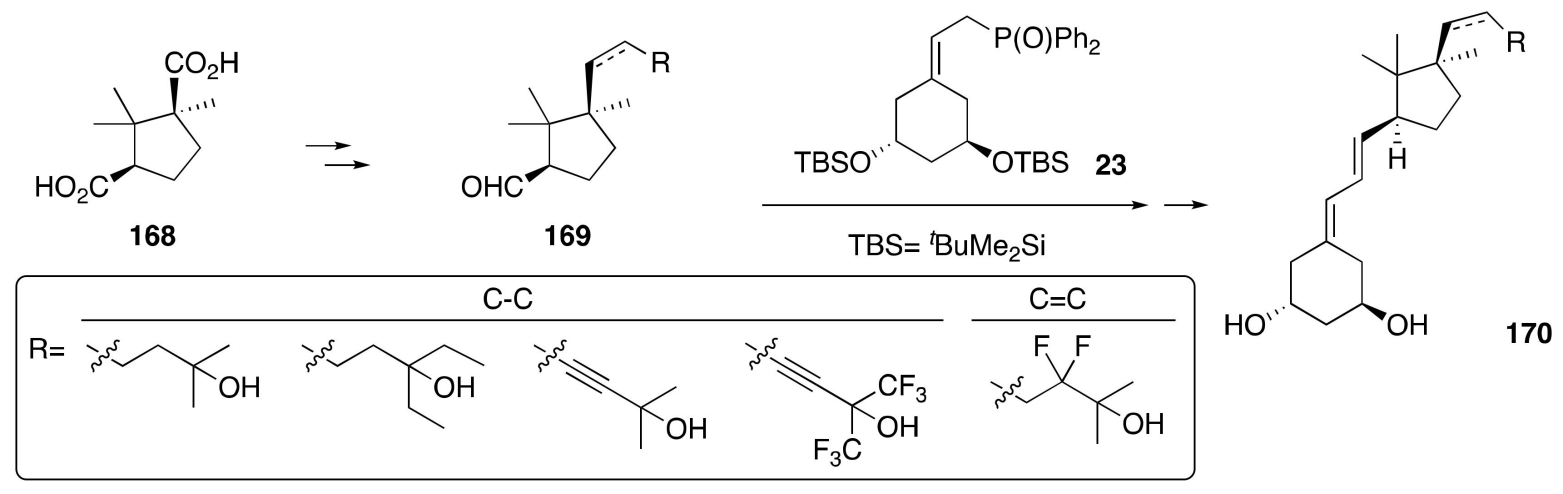
Synthesis of C-ring- and side-chain-modified 1α,25-dihydroxy-19-*nor*-vitamin D_3_.

Vandewalle and co-workers [63] described the synthesis of *seco* C9,11,21-*tri*s*nor*-17-methyl-1α,25-dihydroxyvitamin D_3_ analogs **170** (Scheme 33). These analogs attracted their attention since modifications of that part of vitamin D are the least studied. The structure lacks the six-membered C-ring. To prepare the upper fragment of the molecule, (1*S*,3*R*)-camphoric acid **168** is a good template. A highly efficient two-step differentiation of the carboxylic functions in **168** was attained via silylation of the corresponding diol, affording an aldehyde precursor **169** after several steps. Finally, construction of the title compounds **170** involved Lythgoe coupling of aldehydes **169** with an A-ring phosphine oxide **23**, followed by deprotection of the hydroxy functions. The coupling was performed on D-ring/side chain fragments possessing a free 25-hydroxy function; therefore, an excess of **23** was used, but the A-ring phosphine oxide could be recuperated.

### 5.2. Synthesis of D-Ring- and Side-Chain-Modified Analogs

**Scheme 34:**
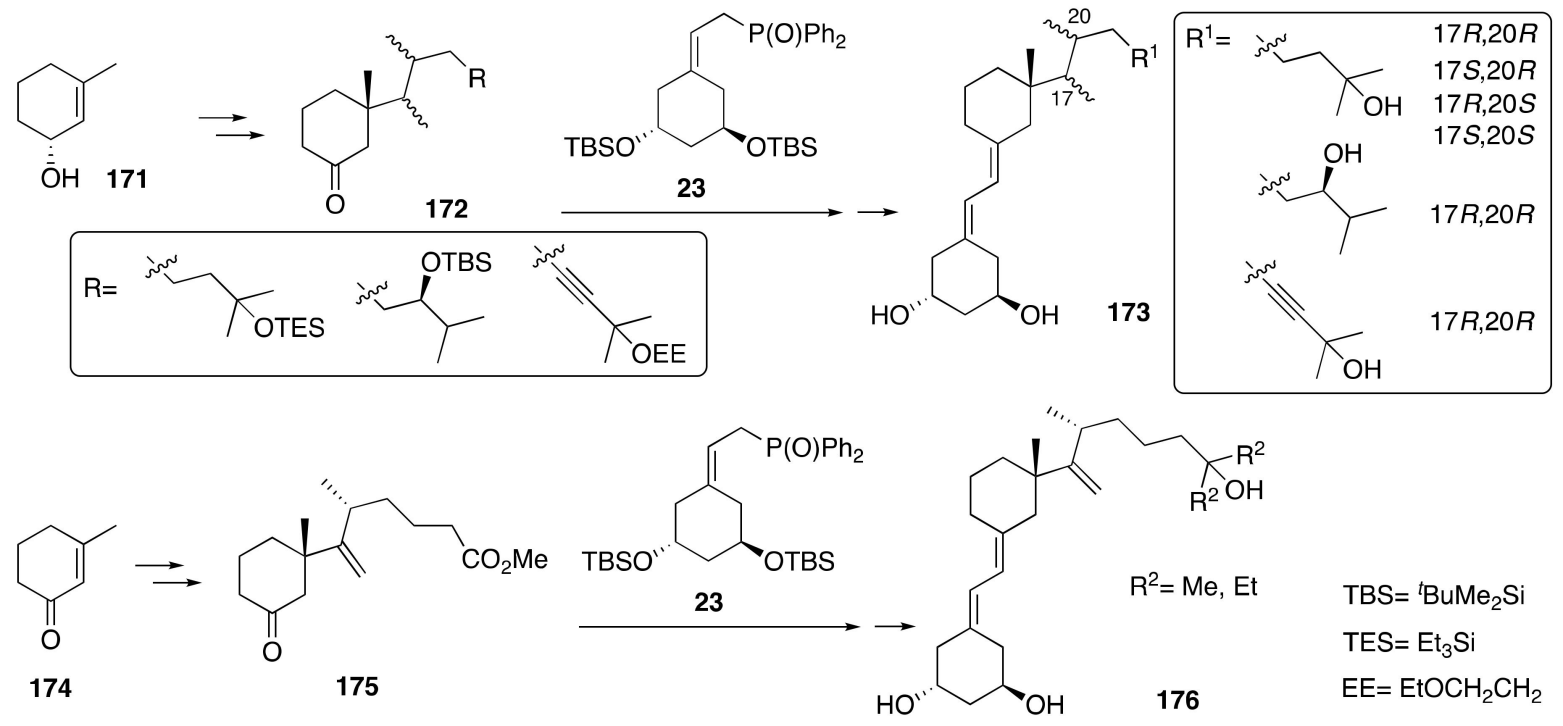
Synthesis of D-ring- and side-chain-modified 1α,25-dihydroxy-19-*nor*-vitamin D_3_.

Several vitamin D analogs **173** and **176** with natural configurations at C17*R* and C20*R* (Scheme 34), which are characterized by the absence of a D-ring and have different side chains, were described by De Clercq’s group [64]. Analogs that lack the closed five-membered ring of the CD-ring skeleton were prepared from (*R*)-3-methyl-2-cyclohexen-1-ol (**171**), which was transformed into the C-ring synthons **172**. In addition, using several reactions, the C-ring moiety **175** was obtained from 3-methyl-2-cyclohexenone (**174**). Cyclohexanone derivatives **172** and **175** were each subjected to the usual Wittig–Horner conditions with a phosphine oxide **23** and, after the removal of the different protecting groups, led to the desired analogs **173** and **176**. The same research group [65] also reported the synthesis of four D-ring-modified, natural side chain 19-*nor*-1α,25-(OH)_2_-D_3_ derivatives **173** lacking C15 and diastereomeric at C17 and C20.

**Scheme 35:**
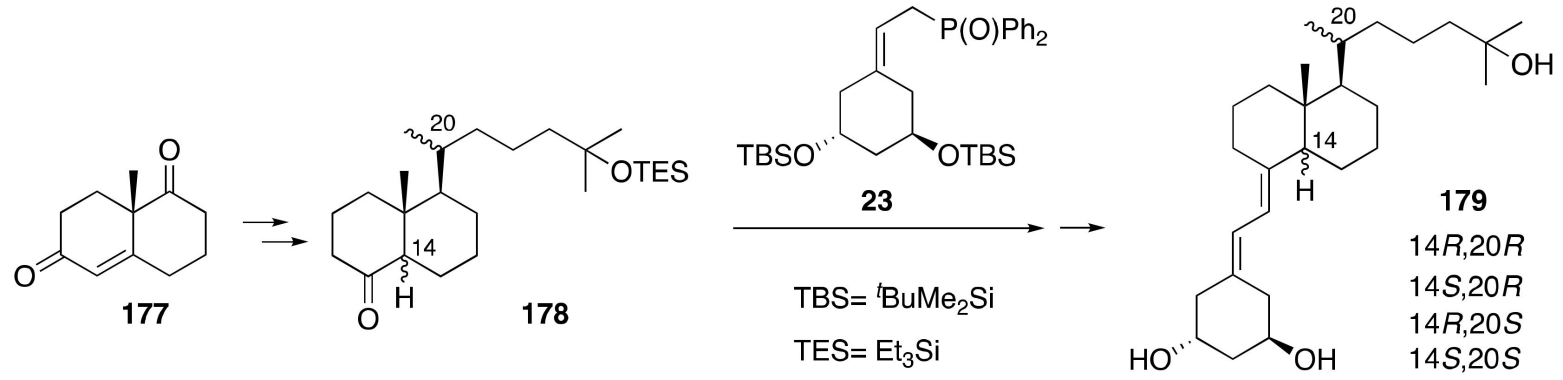
Synthesis of decalin type CD-ring- and side-chain-modified 1α,25-dihydroxy-19-*nor*-vitamin D_3_.

Vandewalle’s group [66,67] presented the synthesis of analogs **179** from a decalin-type CD-ring moiety (Scheme 35). In addition to the fundamental change of the natural hydrindane CD-ring fragment into a decalin system, they also combined this with other modifications as 20-*epi* and/or 14-*epi*, the latter being as consequence of the formation of *trans*- or *cis*-fused decalin. The starting material was the (*S*)-Wieland–Miescher ketone **177**, which, after removal of the keto function, hydroboration of the double bond, and elaboration of the side chain, led to the synthon **178**, ready for coupling. Following that, the Lythgoe coupling of the CD-ring/side chain part **178** and the A-ring synthon precursor **23** gave rise to four different analogs **179**. The yields of the coupling of the *trans*-fused intermediates were lower than that normally observed when the natural hydrindane CD fragment is involved. This could be due to a more severe steric hindrance exerted by the cyclohexane D ring. The reaction of the *cis*-fused decaline precursor produced an even lower yield, again indicating the influence of the nature of the C8 ketone on the Lythgoe coupling.

**Scheme 36:**
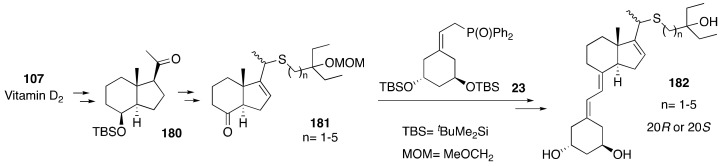
Synthesis of 16-ene-22-thia-19-*nor*-vitamin D_3_ analogs with side chains of different sizes.

Shimizu’s group [68] synthesized eight novel 16-ene-22-thia-26,27-dimethyl-19-*nor*-vitamin D_3_ analogs **182** (Scheme 36) bearing side chains of different sizes in combination with 20*R*- and 20*S*-isomers. The target compounds were prepared by a convergent Wittig–Horner reaction of an A-ring phosphine oxide **23** with 16-ene-22-thia-25-hydroxy Grundmann’s ketones **181** having differently sized side chains, which were derived from a ketone **180**, obtained starting from vitamin D_2_. The biological activities of (20*S*)-22-thia compounds were more potent than those of the corresponding 20*R*-counterparts. Furthermore, the binding affinity of the derivatives **182** to VDR suggest that elongation of the side chain in 22-thia analogs by up to one carbon can be stably accommodated in the VDR binding pocket.

### 5.3. Synthesis of CD-Ring-Modified Analogs

**Scheme 37:**
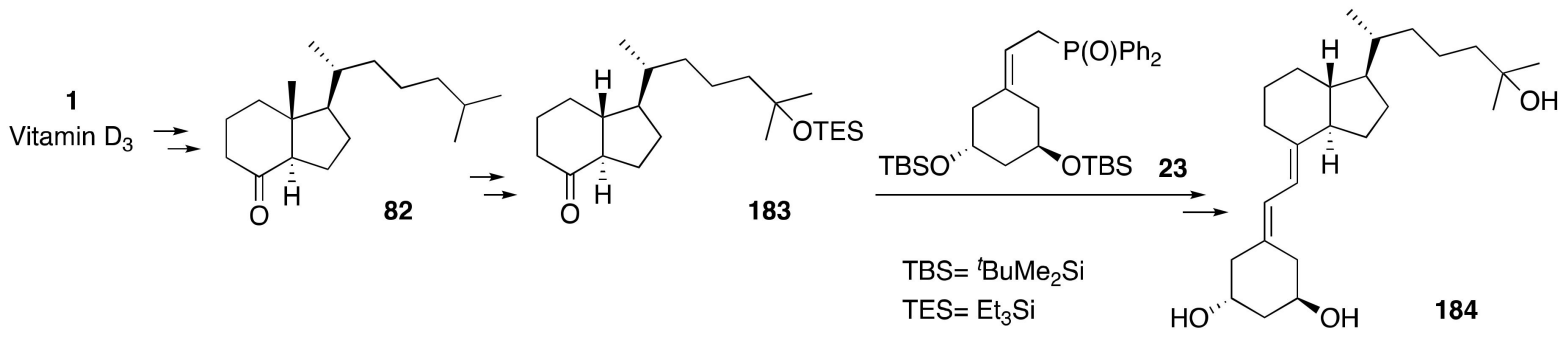
Synthesis of 1α,25-dihydroxy-18,19-*dinor*-vitamin D_3_ analog.

DeLuca and co-workers [69] reported an 18,19-*dinor* analog **184** of 1α,25-(OH)_2_-D_3_ (Scheme 37) to examine the effect of the removal of the 13β-methyl substituent on biological activities. The titled compound was synthesized via Wittig–Horner coupling of a 25-hydroxy-18-*nor* Grundmann type ketone **183** with the corresponding A-ring phosphine oxide **23**. Since the A-ring synthon is known, the attention was focused on the preparation of the appropriate CD-ring fragment, which was obtained from Grundmann’s ketone **82**, which was efficiently prepared by ozonolysis of vitamin D_3_ (**1**). Configuration at C13 in the 18-*nor* Grundmann-type ketone was determined by ^1^H NMR spectroscopy and molecular mechanics calculations. Additional proof of the assigned *trans*-C/D-junction was further confirmed by chemical transformations.

**Scheme 38:**
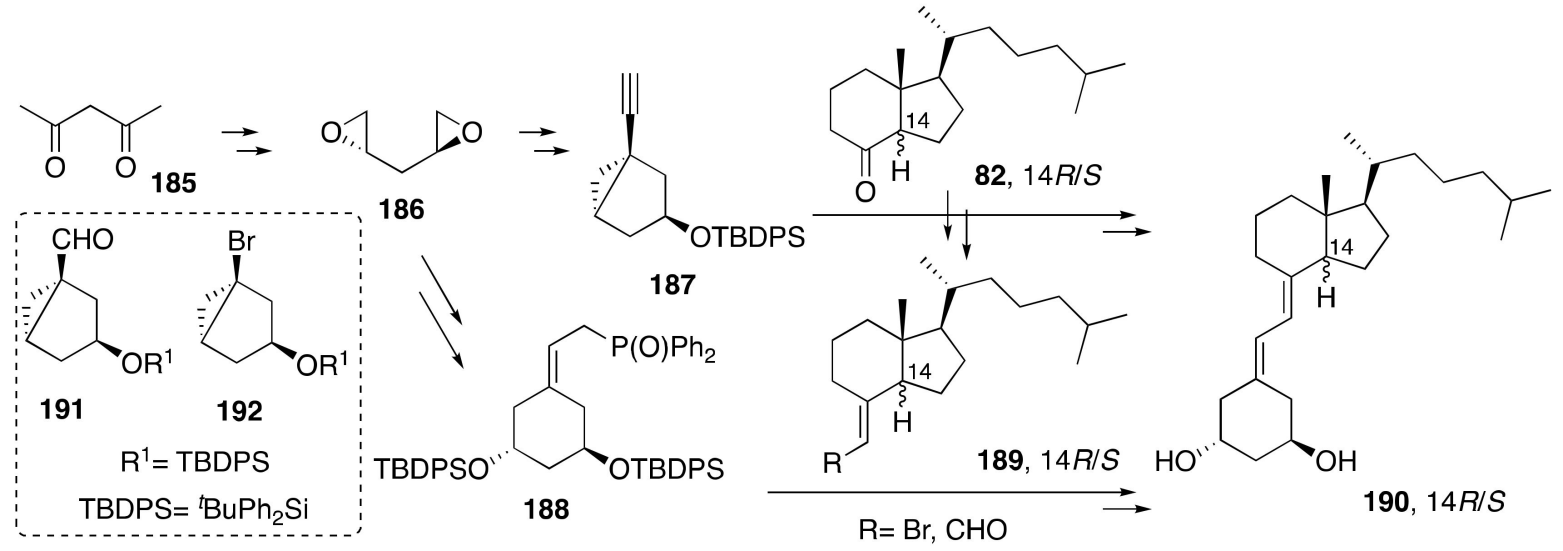
Synthesis of 14-*epi* 1α,25-dihydroxy-19-*nor*-vitamin D_3_.

A convergent route to the A-ring precursors **187** and **188** starting from enantiopure (2*S*,4*S*)-1,2,4,5-diepoxypentane (**186**), which was obtained from 2,4-pentanedione (**185**), was described by Vandewalle’s group (Scheme 38) [70]. Previously, to prepare these A-ring precursors from (−)-quinic acid or from *cis*-1,3,5-cyclohexanetriol, rather linear approaches were involved. A convergent synthesis of A-ring synthons was investigated since the intermediate **186** serves not only to prepare the bicyclic A-ring **187**, but also as a starting point to obtain the phosphine oxide **188**. Because of their interest in 14-*epi* analogs, they synthesized **190** starting from the C/D *cis*-fused **82**. They also developed an alternative route to **190** involving the coupling of an aldehyde **191** with the vinylic lithium derivative obtained from **189a** (R = Br). In addition, they reported [71] a new synthesis for the A-ring precursor **192** from the known 3-cyclopentenol and applied it to the synthesis of **190** upon the reaction of the lithiated intermediate prepared from **192** with the aldehyde **189b** (R = CHO).

### 5.4. Synthesis of CD-Ring- and Side-Chain-Modified Analogs

**Scheme 39:**
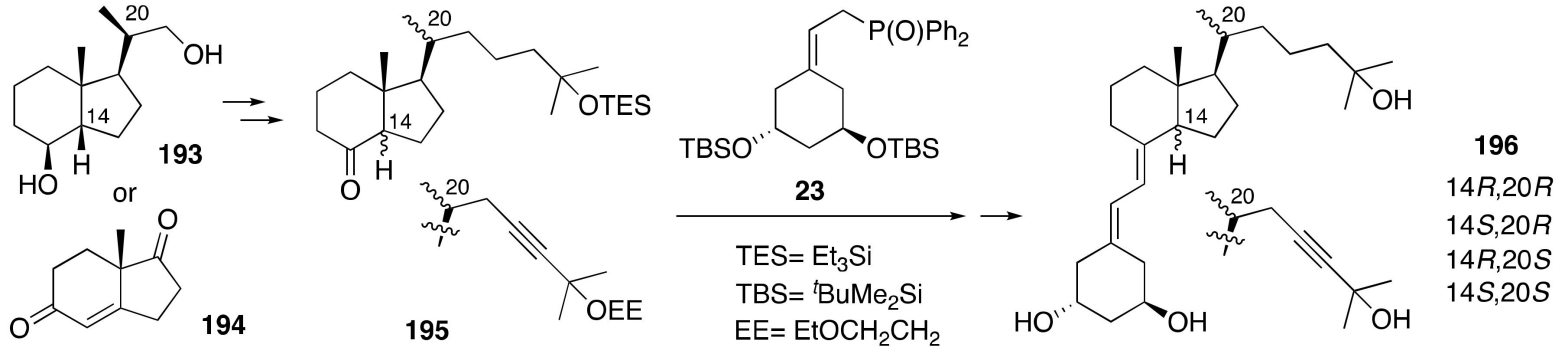
Synthesis of 14,20-bis-*epi* 1α,25-dihydroxy-19-*nor*-vitamin D_3_.

Vandewalle and co-workers [72] described the synthesis of 14,20-bis-*epi*-1α,25-dihydroxy-19-*nor*-vitamin D_3_ and side chain analogs **196** (Scheme 39). The syntheses of CD-ring precursors **195** were performed through the degradation of vitamin D_2_ via the 14,20-bis-*epi*-Inhoffen–Lythgoe diol **193** and by total synthesis starting from the Hajos–Wiechert ketone **194**. The CD-ring/side chain precursors **195** were coupled with a 19-*nor*-A-ring precursor **23** using the Wittig–Horner olefination reaction.

**Scheme 40:**
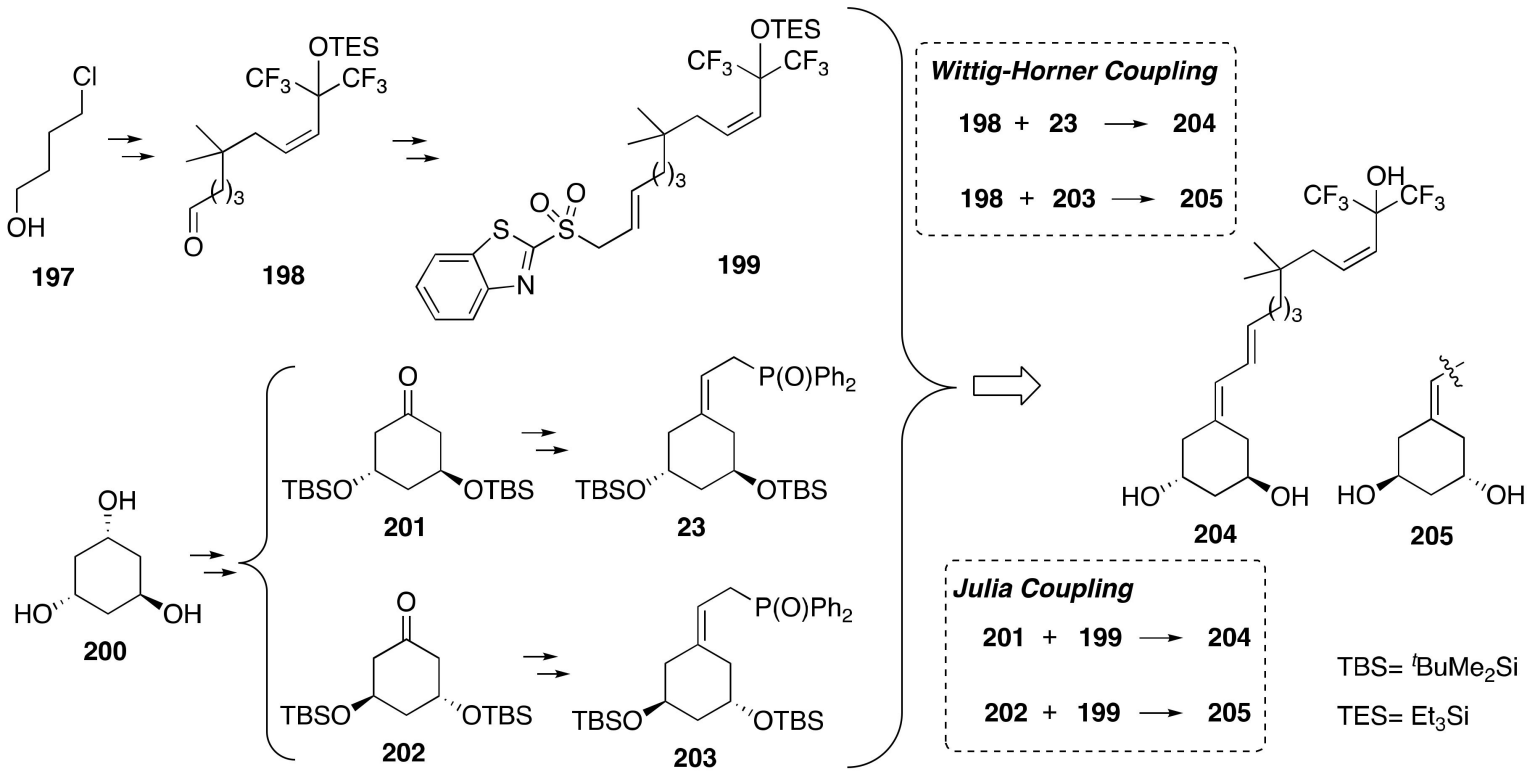
Synthesis of *des*-C,D 1α,25-dihydroxy-19-*nor*-vitamin D_3_.

Retiferol (**204**, Scheme 40) was selected as a clinical candidate for the evaluation of a potential oral therapy for psoriasis since it is a potent activator of the vitamin D receptor with low calcemic activity. A short and efficient route to the *des*-C,D vitamin D_3_ derivative **204** and its enantiomer **205** was described by Hilpert and Wirz [73]. This route features an assembly strategy using a modified Julia olefination of the A-ring ketones **201**/**202** and the 2-benzothiazolyl sulfone **199**. A three-step procedure from the *meso trans*-1,3,5-cyclohexane triol (**200**), which involves an enzymatic desymmetrization, was used to prepare the A-ring synthon precursor in the case of analog **204**. New routes to the aldehyde **198**, prepared from 4-chlorobutanol (**197**), and to the phosphine oxide **23** and its enantiomer **203** were developed. Alternative coupling strategies (Wittig–Horner or Julia olefinations) were also used. Similarly, the preparation of both enantiomers of the phosphine oxides **23** and **203** was based on chemoenzymatic routes starting from *meso*-1,3,5-trihydroxy cyclohexane derivatives **200** and *cis*-1,3,5-cyclohexanetriol (**19**, Scheme 3), respectively. From a variety of assembly strategies evaluated, the uncommon disconnection, accomplished via a modified Julia olefination involving the ketone **201** and the sulfone **199**, emerged as the most efficient strategy, resulting in a new short and high-yielding route to the retiferol (**204**).

**Scheme 41:**
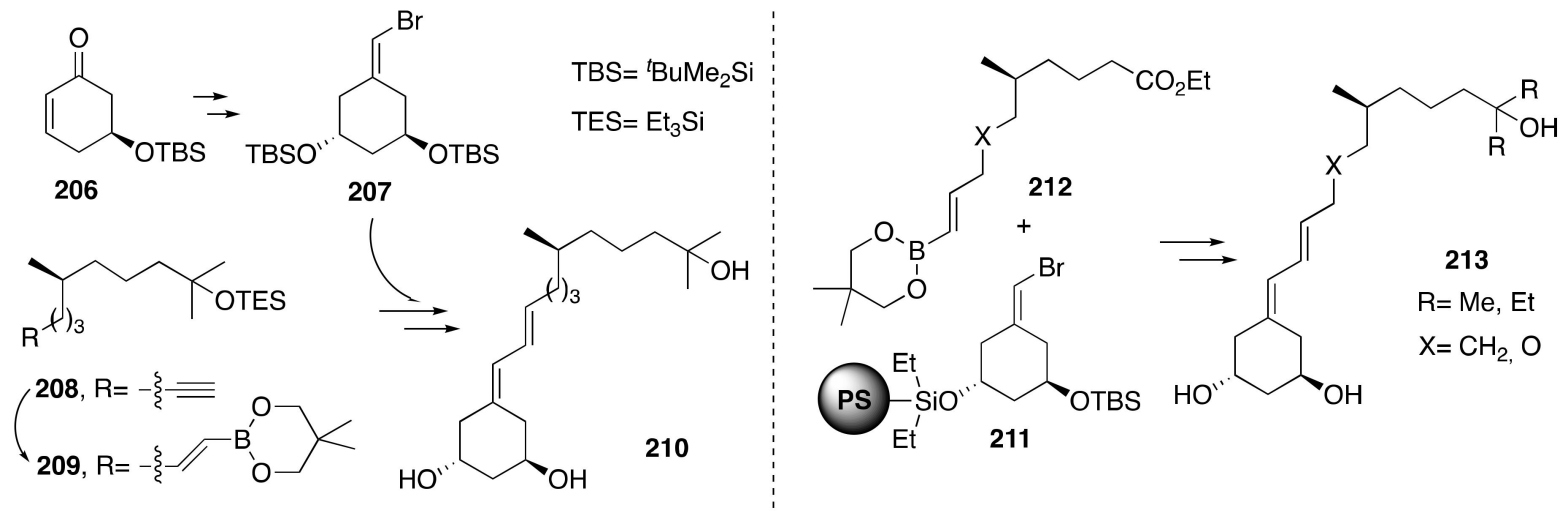
Synthesis of *des*-C,D 1α,25-dihydroxy-19-*nor*-vitamin D_3_ in solution and on a solid support.

Sato and co-workers [74] reported an efficient and high-yield preparation of **207** (Scheme 41) and its utilization for the synthesis of *des*-C,D derivatives of 19-*nor*-1α,25-dihydroxyvitamin D_3_, including **210** and **213**, by the Suzuki–Miyaura coupling. The A-ring intermediate **207**, which was efficiently prepared from readily available 5-(*tert*-butyldimethylsilyl)oxycyclohex-2-enone (**206**) that came from (*S*)-epichlorohydrine, reacted with the boronate compound of the C,D-ring portion **209**. The latter was prepared by hydroboration of acetylene **208**. The method was also applied to a solid-phase synthesis, and the A-ring synthon **211** was easily prepared on a solid support from an intermediate of the synthesis of **207**. The coupling of the polymer-bounded A-ring synthon precursor **211** with *des*-C,D/side chain moieties **212** was the key step to generate the analogs **213**. The solid support synthesis could be applied to the fields of drug discovery and manufacturing.

## 6. Synthesis of Side-Chain-Modified 1α,25-(OH)_2_-19-*nor*-Vitamin D

**Scheme 42:**
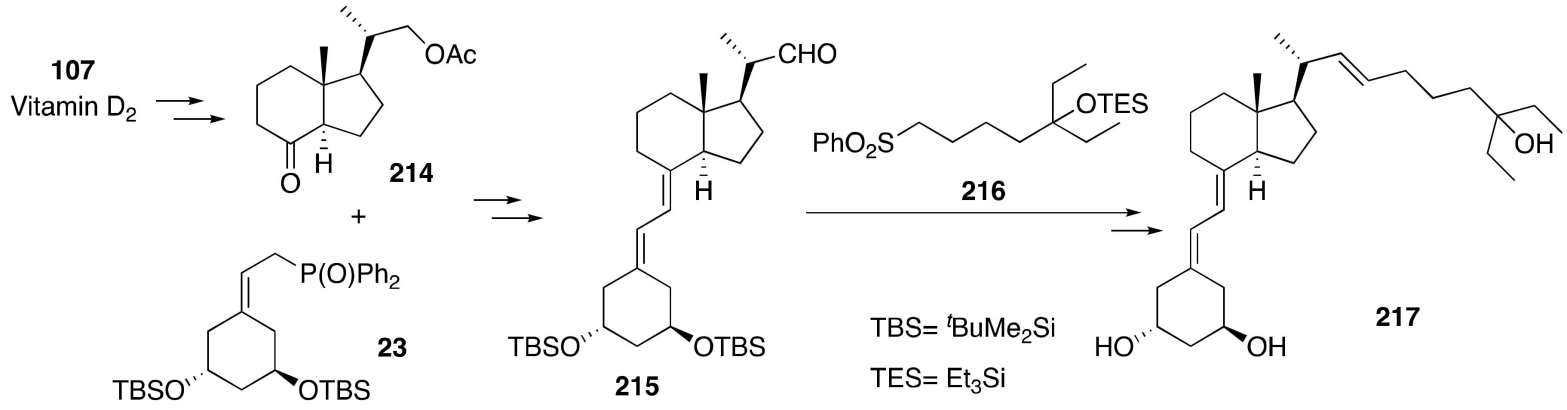
Synthesis of Δ^22^,22*E*-24,24-dihomo-26,27-dihomo-1α,25-dihydroxy-19-*nor*-vitamin D_3_.

With the aim to examine the biological effect of side chain modifications of 19-*nor*-vitamin D derivatives, Perlman and DeLuca [75] described a side-chain-homologated lα,25-dihydroxy-19-*nor*-vitamin D analog **217** (Scheme 42), which was prepared in a double convergent synthesis with 1α-hydroxy-19-*nor*-vitamin D C22 aldehyde **215** as a key intermediate. This aldehyde was prepared in a convergent synthesis from the A-ring synthon **23** obtained from commercially available quinic acid. The readily available Inhoffen–Lythgoe diol served as the basic building block and was obtained from the oxidation of vitamin D_2_ (**107**), providing the CD ring acetylated moiety **214**. Condensation of the A-ring synthon **23** with the ketone **214** in a Wittig–Horner reaction gave the diene **215**. Then, this intermediate was used as a precursor to synthesize Δ^22^,22*E*,19-*nor*-24,24-dihomo-26,27-dihomo analog **217** via Julia olefination of the 19-*nor*-22-aldehyde **215** with the corresponding protected phenylsulfone side chain fragment **216**.

**Scheme 43:**
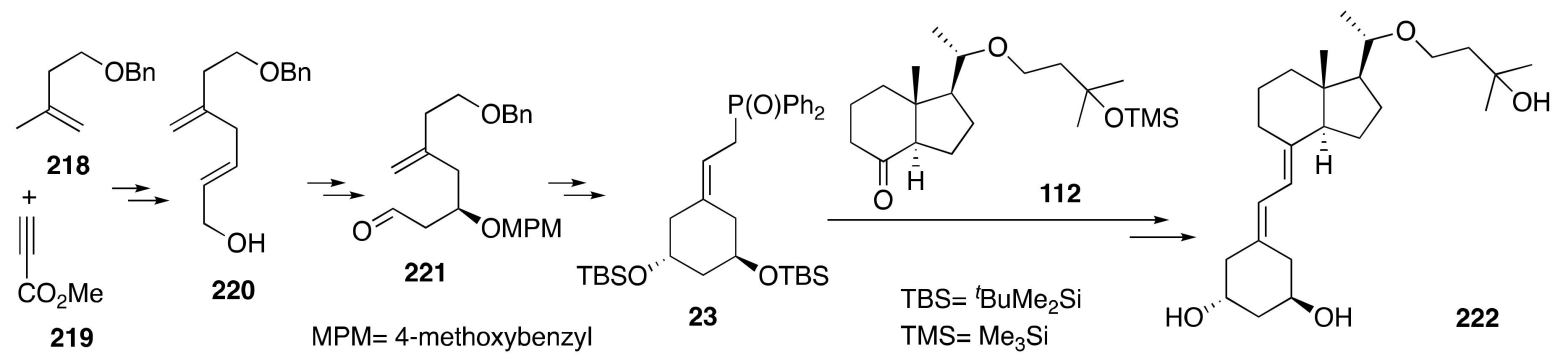
Synthesis of 22-oxa-1α,25-dihydroxy-19-*nor*-vitamin D_3_ analog.

Mikami, Okano, and co-workers [76] reported the synthesis of a hybrid 22-oxa-1α,25-dihydroxy-19-*nor*-vitamin D_3_ analog **222** (Scheme 43) through Wittig–Horner olefination between the CD-ring/side chain fragment **112** and the A-ring synthon precursor **23**. The ene-cyclization substrate (*R*)-**211** was prepared via catalytic enantioselective epoxidation of allylic alcohol **220**, which was obtained through a regioselective propiolate-ene reaction of homoallylic ether **218** with methyl propiolate (**219**). Thus, the combination of the regioselective propiolate-ene reaction, catalytic enantioselective epoxidation, and catalytic enantioselective carbonyl-ene cyclization allowed access to the A-ring synthon precursor **23** and the analog **222**.

**Scheme 44:**
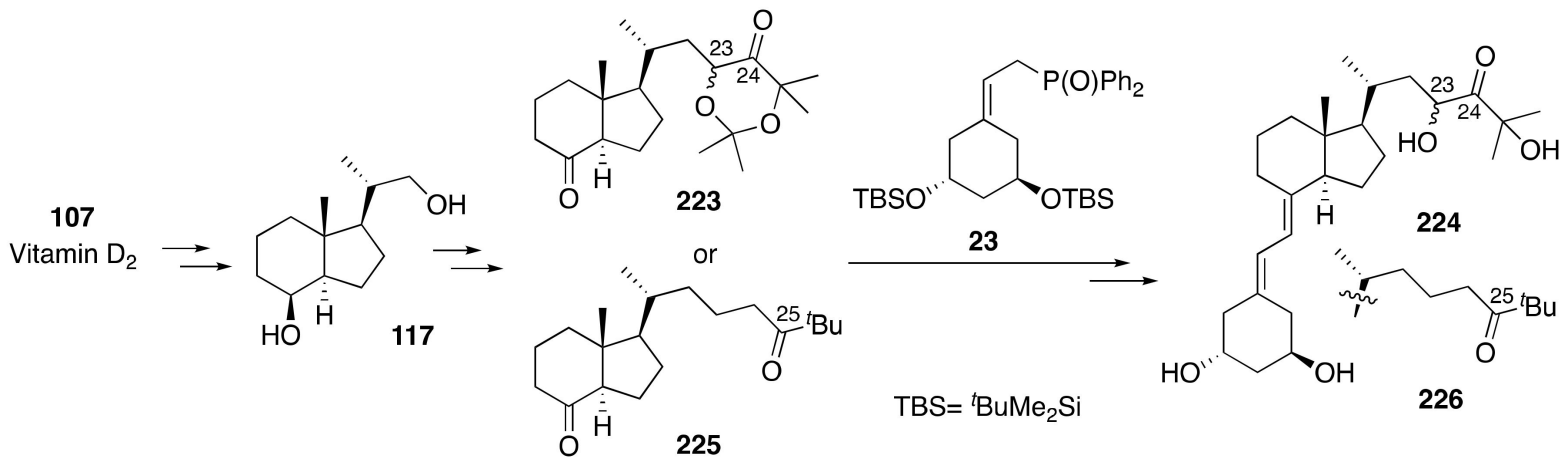
Synthesis of hydroxy ketones and ketone derivatives of 1α,25-(OH)_2_-19-*nor*-vitamin D_3_.

In a previous report, Reddy’s group indicated that 1α,23(*S*),25-trihydroxy-24-oxovitamin D_3_, a natural metabolite of 1α,25-dihydroxyvitamin D_3_, is almost equipotent to 1α,25-(OH)_2_-D_3_ in suppressing parathyroid hormone (PTH) secretion in addition to possessing only weak in vivo calcemic actions. In an attempt to produce vitamin D_3_ analogs with a better therapeutic index, Reddy and co-workers [77] synthesized C23 epimers of 1α,23,25-(OH)_3_-24-oxo-19-*nor*-vitamin D_3_ **224** (Scheme 44) by coupling the A-ring phosphine oxide **23** with CD-ring ketones **223** using the Horner–Wittig method. The 19-*nor* A-ring precursor **23** was prepared from quinic acid. The appropriate CD-ring ketones **223** were synthesized from Inhoffen–Lythgoe’s diol **117**, which was obtained from oxidation of vitamin D_2_ (**107**). 1α,23(*R*),25-(OH)_3_-24-oxo-19-*nor*-D_3_ **224a** and, to a lesser extent, its 23(*S*) diastereomer **224b** were potent analogs, and they suppressed PTH secretion in bovine parathyroid cells and strongly inhibited clonal growth and induced differentiation of HL-60 cells in vitro. Using a similar approach, Posner and co-workers [78] reported a new side-chain ketone analog **226** from a diketone **225**. Surprisingly, the ketone analog 19-*nor*-**226** was not significantly less calcemic in vivo than the corresponding 19-methylene derivative, according to DeLuca’s observations that 19-*nor* analogs are usually much less calcemic than their 19-methylene versions.

**Scheme 45:**
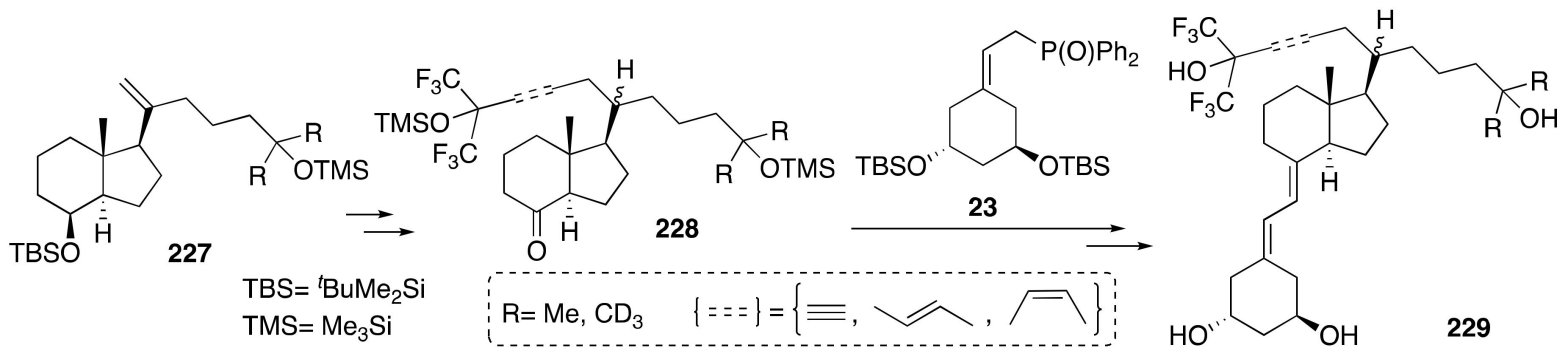
Synthesis of 1α,25-dihydroxy-19-*nor*-vitamin D_3_ Gemini analogs.

Maehr’s research group described [79,80] new Gemini analogs **229** (Scheme 45), which consisted of derivatives with two different side chains emanating from C20. The first part of the syntheses involved the preparation of CD-ring synthons and commenced with the previously described Inhoffen–Lythgoe diol, which was transformed into compounds **227**. Subsequent reactions led to the ketones **228**, which served as suitable coupling partners with the A-ring synthon **23**. The second phase of the synthesis included the coupling reactions of the CD-ring moieties **228** with the A-ring precursor **23** using Wittig–Horner conditions. These analogs, featuring 23-yne and 23(*E*) side-chains, were more active in human breast cancer cell growth inhibition and human leukemia cell differentiation induction than their 23(*Z*)-counterparts.

**Scheme 46:**
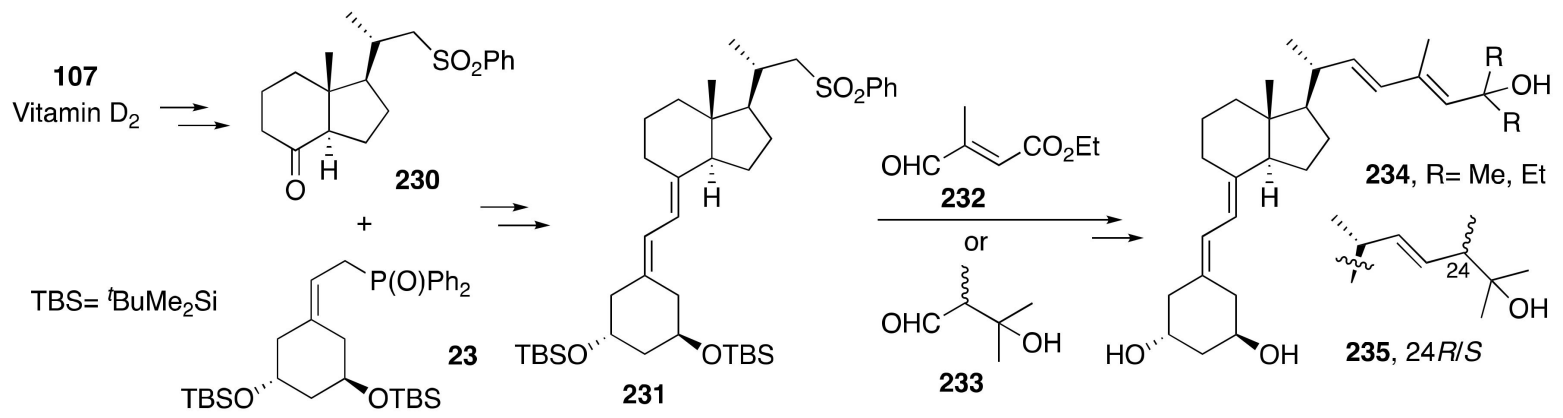
Synthesis of 1α,25-dihydroxy-19-*nor*-vitamin D_2_ analogs.

The analogs of 19-*nor*-1,25-(OH)_2_-D_2_ **234** (Scheme 46) with side chain modifications were synthesized by Pietraszek’s group [81] using a convergent strategy with the new advanced intermediates **230** and **23**. The design of this synthesis was developed while taking into account its possible scale-up for pharmaceutical purposes. Thus, starting from commercially available compounds, vitamin D_2_ and quinic acid, the synthesis proceeded through convenient bench-stable intermediates. The Wittig–Horner condensation of the phosphine oxide **23** with the ketone **230** generated the new sulfone **231** as the key intermediate in the synthesis of the title compounds. Julia olefination of the deprotonated sulfone **231** with the aldehydes **232** or **233** gave the corresponding hydroxysulfones. In the first case, the mixture was reacted with a Grignard reagent to give the respective tertiary alcohols, and then radical dehydroxy-desulfonylation followed by desilylation produced the target analogs **234a** (R = Me) and **234b** (R = Et), respectively. The synthesis of the side chain fragments of paricalcitol **235a** and its (24*R*)-diastereomer **235b** started from the homochiral methyl (*S*)- and (*R*)-3-hydroxy-2-methylpropionate, respectively. The Grignard reaction of these esters, followed by the Dess–Martin oxidation of the resulting crude diols, yielded the aldehydes **233a** and **233b**, respectively.

## 7. Concluding Remarks

Nearly 50 years have passed since the discovery of calcitriol (1α,25-dihydroxyvitamin D_3_), the hormonally active form of vitamin D. However, the field of vitamin D research continues to progress, and a number of review articles have appeared that describe several aspects of the vitamin D endocrine system since this hormone modulates numerous physiological functions beyond mineral metabolism. Many hundreds of vitamin D analogs have been synthesized in an attempt to segregate these two opposite effects. A special class of analogs, 19-*nor*-vitamin D, has been demonstrated to have lower calcemic effects than the natural hormone, maintaining or even increasing the other regulatory activities. As a consequence, a huge effort has been made in relation to the synthesis of new 19-*nor*-vitamin D derivatives. It is well established that the structural characteristics of the vitamin D analogs play an important role in their mode of action. Therefore, understanding the molecular basis of vitamin D action at the molecular level will be crucial in order to design new analogs. The knowledge accumulated from studies on different derivatives will enable the design of a new generation of vitamin D analogs with enhanced selectivity. Sometimes, the new derivatives are hybrids, which means that they have more than a single modification in one or more parts within the molecule. The present review provides primary references in the literature that, in the past three decades, have reported the preparation of 19-*nor* modified vitamin D analogs, which could be interesting to the synthetic chemist as well as to other scientists in related fields who wish to understand the chemical strategies leading to new vitamin D analogs with useful pharmacological properties of biomedical interest. The details of the behavior of vitamin D at the molecular level are emerging, but they are still far from complete. It is foreseeable that studies in the near future will continue to develop the design and synthesis of new derivatives. Further research is encouraged in this exciting area, which could yield important therapeutic improvements in the medium-short term.
